# Peripheral nerve stimulation for lower‐limb postoperative recovery: A systematic review and meta‐analysis of randomized controlled trials

**DOI:** 10.1002/pchj.794

**Published:** 2024-09-16

**Authors:** Jingxinmiao Lin, Dong Song, Yiheng Tu, Huijuan Zhang

**Affiliations:** ^1^ CAS Key Laboratory of Mental Health Institute of Psychology, Chinese Academy of Sciences Beijing China; ^2^ Department of Psychology University of Chinese Academy of Sciences Beijing China; ^3^ Department of Neurology The First Affiliated Hospital of Zhengzhou University Zhengzhou China

**Keywords:** functional improvements, lower‐limb postoperative recovery, meta‐analysis, pain relief, peripheral nerve stimulation (PNS)

## Abstract

Patients undergoing lower‐limb orthopedic surgery may experience multiple postoperative complications. Although peripheral nerve stimulation (PNS) is a promising non‐pharmacological approach that has been used in lower‐limb postoperative recovery, the clinical efficacy of PNS remains inconclusive. This study systematically searched three databases (PubMed, Embase, and Cochrane Library) for randomized controlled trials (RCTs) that examined the treatment effects of PNSs in patients who underwent lower‐limb orthopedic surgery up to September 29, 2023. Two investigators independently identified studies, extracted data, and conducted meta‐analyses with Review Manager 5.4. The outcomes were pain relief (measured by reductions in pain intensity and analgesic consumption) and functional improvements (range of motion [ROM] and length of hospitalization [LOH]). A total of 633 patients including 321 in the experimental groups and 312 in the control groups from eight RCTs were included. PNS showed no significant effect on pain intensity, while analgesic consumption was marginally significantly reduced in the experimental group. Furthermore, no significant differences were observed regarding functional improvements in ROM or LOH after the intervention. Although PNS had no significant effect on pain relief or functional improvements, the intervention exhibited a marginally significant reduction in analgesic consumption. Future trials should be conducted with larger sample sizes, longer follow‐up periods, and more varied stimulation parameters.

## INTRODUCTION

With the increase in the aging population (World Health Organization, 2023), the number of individuals at high risk for falls with fractures will double by 2040, reaching 319 million (Odén et al., 2015). Orthopedic surgery is commonly viewed as an efficacious clinical treatment for fracture (Blom et al., 2021). Patients undergoing orthopedic surgery due to fractures may experience multiple postoperative complications, including unexpectedly returning sepsis (42%), failure to wean from the ventilator (31%), and organ surgical site infections (27%) (Tevis & Kennedy, 2016). These complications might prolong the length of the hospital stay and increase the incidence of dispositions to institutional care facilities after surgeries (Khan et al., 2006; Legner et al., 2009). Importantly, orthopedic surgery is typically associated with a high proportion of moderate to severe postoperative pain, which negatively impacts the patient's quality of life and can lead to additional complications, such as pressure sores and muscle atrophy, and increase morbidity and mortality risk (Lovich‐Sapola et al., 2015; Skirven & Trope, 1994). Opioids are often prescribed for postoperative pain management (Kehlet, 1997). However, using these medications for postoperative pain management may cause multiple adverse effects, such as dizziness, nausea, vomiting, tolerance, constipation, physical dependence, respiratory depression, and increased addiction risk (Benyamin et al., 2008; Gabriel et al., 1991; Scheiman et al., 2006; Wolfe et al., 1999). Non‐pharmacological approaches have been introduced as options for clinical pain management, such as physical therapy, i.e. peripheral nerve stimulation (PNS) (Archer et al., 2013; Cho et al., 2023; Geneen et al., 2017; Goroszeniuk & Pang, 2014; Jette et al., 2020; Wang et al., 2022; Xu et al., 2021).

Neuromodulation can modulate nerve activity through the targeted delivery of a stimulus, such as electrical or chemical agents, altering nerve networks in the body (Knotkova et al., 2021). In particular, PNSs, including invasive approaches (e.g., percutaneous PNS [PPNS]) and noninvasive approaches (e.g., nerve electrical stimulation [NES], electrical stimulation [ES], transcutaneous electrical acupoint stimulation [TEAS], transcutaneous electrical nerve stimulation [TENS], transcutaneous electrical stimulation [TES]), have been widely applied to relieve pain and promote functional recovery after orthopedic surgery (Arvidsson & Eriksson, 1986; Avramidis et al., 2003; Ilfeld et al., 2019; Stevens‐Lapsley et al., 2012; Wang et al., 2022). However, the efficacy of PNS in lower‐limb postoperative rehabilitation remains uncertain. Some studies have shown that PNSs exert significant therapeutic effects in reducing postoperative pain (Ilfeld et al., 2017, 2021), while others have indicated no significant difference in decreasing pain rating compared to the control groups (Johnson et al., 2015; Khadilkar et al., 2008). This inconsistency may arise from various complicating factors, such as the specific stimulation parameters (e.g., frequency, intensity, targeted position, and duration) and individual differences. To the best of our knowledge, there is no systematic review incorporating a meta‐analysis of large sample randomized controlled trials (RCTs) to investigate the impact of PNS treatments on reducing pain intensity, analgesic consumption, and functional impairments in lower‐limb postoperative patients. This deficiency of high‐quality synthesized evidence has constrained the clinical translation and implementation of such findings. The present study aims to address this gap by conducting a meta‐analysis of the available data to evaluate the effect of PNSs on lower‐limb postoperative rehabilitation. An integrated evaluation methodology encompassing risk of bias evaluation, heterogeneity assessment, evidence quality grading and sensitivity analysis will provide valuable insights into the clinical effectiveness of PNS, particularly in elucidating parameter variability often observed in traditionally underpowered small‐sample studies.

Therefore, we conducted a systematic review and meta‐analysis of RCTs to evaluate the efficacy of PNS in rehabilitation for patients after lower limb surgery. The outcome parameters considered to assess the efficacy of the treatment included pain relief (i.e., the reductions of pain intensity and analgesic consumption) and functional improvements (i.e., the increase in range of motion [ROM] and decrease in length of hospitalization [LOH]). This systematic evaluation to establish an evidence base for PNS efficacy may provide a theoretical basis and potential guidance for the subsequent clinical applications of PNS.

## METHODS

The study protocol was submitted to PROSPERO (International Prospective Register of Systematic Reviews in Health and Social Care) on June 4, 2022, and registered on August 3, 2022 (ID: CRD42022336815). This study followed the 2020 Preferred Reporting Items for Systematic Reviews and Meta‐Analysis guidelines for reporting systematic reviews and meta‐analyses (Page et al., 2021), and was performed with a thorough assessment using the PRISMA (Preferred Reporting Items for Systematic Reviews and Meta‐Analyses) and AMSTAR (A MeaSurement Tool to Assess systematic Reviews) checklists (Supplementary Files 1 and 2) (Shea et al., 2017).

### Eligibility criteria and search strategy

Data were extracted from PubMed (https://pubmed.ncbi.nlm.nih.gov), Embase (https://www.embase.com), and the Cochrane Library database (https://www.cochranelibrary.com) on September 29, 2023. The study selection process was performed based on the PICOS framework principles (Moher et al., 2009), which included the following (details are shown in Supplementary File 3): population (P): patients who underwent lower‐limb orthopedic surgery; intervention (I): PNS, including TENS, PPNS, NES, ES, TEAS, and TES; comparison (C): the differences between the experimental group (received real PNS in postoperative rehabilitation) and the control group (comprised subjects who did not receive PNS and instead were administered either a placebo intervention or sham stimulation after lower limb orthopedic surgery); outcome (O): the primary outcomes were pain intensity and analgesic consumption, while the secondary outcomes were ROM and LOH; and study design (S): RCTs.

**FIGURE 1:**
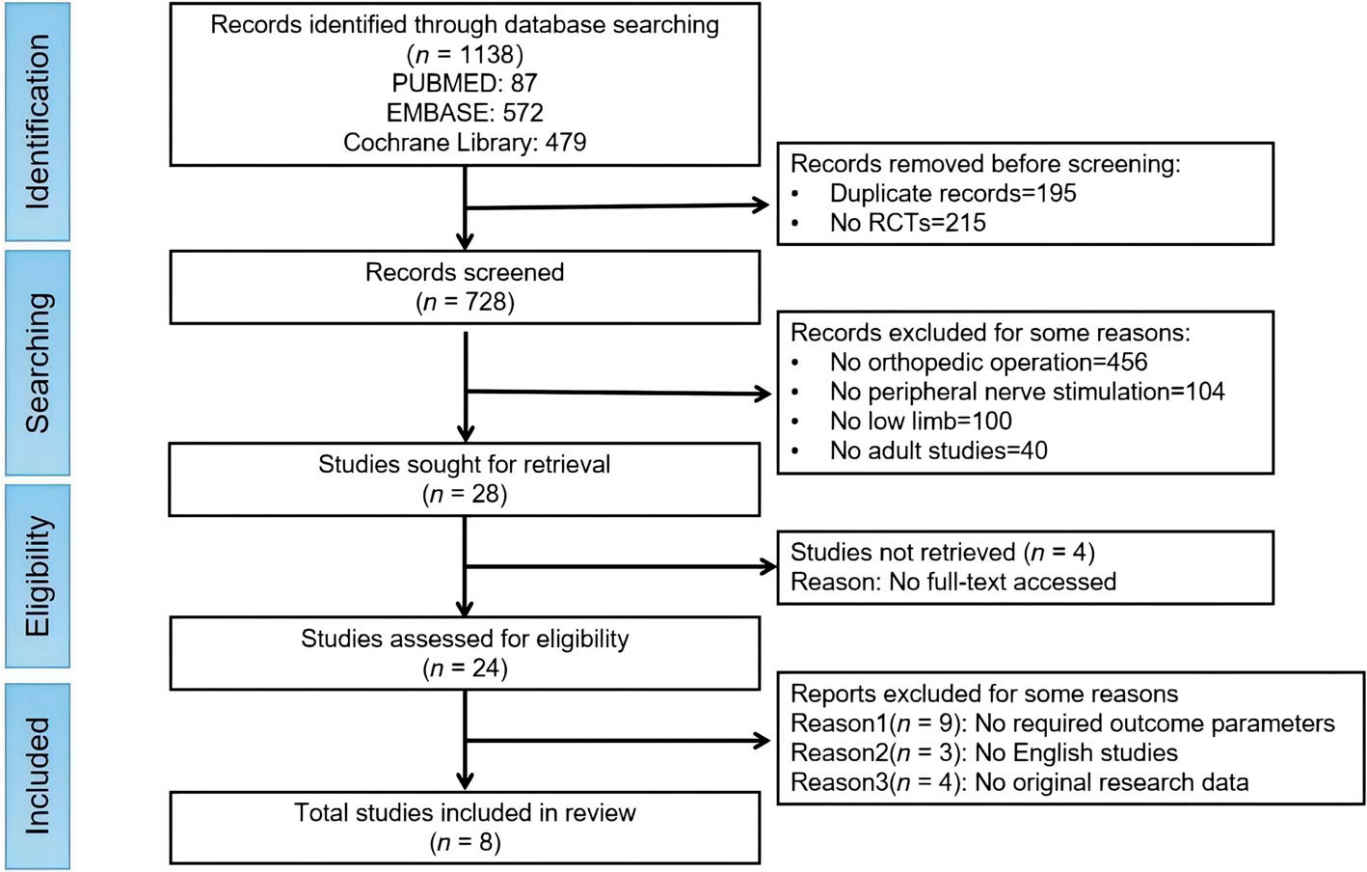
PRISMA flow diagram of the articles through the study selection process.

Peer‐reviewed, full‐text, and English‐language publications were considered eligible for inclusion in the systematic review, provided they met the following criteria: (1) participants were at least 18 years of age when scheduled to undergo lower‐limb orthopedic surgery; (2) studies were RCTs; (3) participants were able to understand and consent to participate in the studies; (4) the experimental groups received at least one PNS therapy; and (5) the control group included at least one negative control, such as a sham control, placebo, or medication‐only treatment. The exclusion criteria for this study are presented in the screening flowchart (Figure 1). Apart from excluding duplicate and non‐RCTs articles (review, protocol, and guideline, etc.), the main exclusion criteria included (1) non‐orthopedic postoperative cases; (2) non‐PNS as primary intervention; (3) non‐lower limb affected sites; (4) non‐adult studies; (5) non‐English language publications; (6) unable to access the full text; (7) lack of key outcome parameters and original research data.

The search strategy combined index terms with limits and filters in accordance with the principle of PICOS (Supplementary File 3). At the beginning, we identified index in each database (MEDLINE's medical subject headings [Mesh Terms] in PubMed and Cochrane Central Register of Controlled Trials, and Emtree in Embase), and searched the “Entry Terms” or subheadings to identify all synonyms and variants of related phrases to expand the search scope and find more studies. The various search parts of PICOS were completed with each index and phrase combined syntax (archive code, adjacency search, Boolean operators, truncation, etc.). For PubMed, Embase and Cochrane Central Register of Controlled Trials search, we added a best balance of sensitivity and specificity filter for identifying randomized trials developed by the McMaster University of Health Information Research Unit (https://hiruweb.mcmaster.ca/hkr/hedges/medline/; https://hiruweb.mcmaster.ca/hkr/hedges/embase/). Reference lists of included articles were manually screened to identify additional studies. The approach to study identification from this systematic review is transparently reported in the Supplementary File 4.

### Selection process

A two‐step screening process to identify the final studies to be included in the meta‐analysis was employed. During the process of searches, two reviewers (J.L. and D.S.) collaboratively developed the search strategies and then independently performed the literature searches. This involved removing duplicates, screening titles and abstracts, reviewing full texts, and compiling the final list of included studies.

In the first step, the two reviewers screened the title and abstract of each article using the exclusion criteria to eliminate unsuitable citations. In the second step, they obtained the full text of the remaining citations and assessed them to determine whether they met all the inclusion criteria. Following completion of the searches, J.L. and D.S. engaged in peer review by cross‐checking a random sample of the search results to ensure accuracy and comprehensive identification of relevant literature. The reviewers consulted with each other to make the final determination if there was any uncertainty regarding the eligibility of a study. Any discrepancies between the reviewers' selection of studies were discussed until a consensus was reached. For the portions that could not be conclusively determined, H.Z. and Y.T. made determinations based on the inclusion and exclusion criteria. Additionally, the reference lists of relevant studies were manually searched to identify any additional studies that met the inclusion criteria.

### Data collection

Two reviewers (J.L. and D.S.) independently extracted data from the included RCTs and checked for data consistency before entering them into the software. Disagreement was resolved by consensus, with reviewers (Y.T. and H.Z.) acting as arbiters. Subsequently, data were extracted from the eligible studies, including the first authors, published dates, effects, interventions, indications, country, groups, age, sex, and main outcome parameters.

After the final screening, pain relief and functional improvement were extracted from the eligible studies as outcome measurements for further statistical analysis. The primary outcomes were the differences in pain intensity and changes in analgesic consumption between the experimental and control groups after the intervention. Postoperative orthopedic rehabilitation is usually initiated in the acute and subacute phases when the patient's postoperative condition has stabilized. The intervention time to initiate PNS was defined as within 24 hours after surgery. Pain relief was subjectively assessed using multiple pain scales, such as the visual analog scale (VAS), numeric rating scale (NRS), brief pain inventory (BPI), and quality of recovery‐40 (QoR‐40), with 0 indicating no pain and the maximum number representing severe pain. Additionally, analgesic or anesthetic drug consumption served as an objective indicator. The secondary outcomes included differences in limb functional improvements measured by ROM and LOH between the groups after the intervention. ROM, one of the functional indicators, was used to show the difference in the affected joint, which was measured by a handheld goniometer to assess both the active and passive ROM following the principles of rehabilitation assessment. A minority of the outcomes of included RCTs (three out of eight) measured multiple sessions of PNS, primarily including post‐discharge follow‐up assessments. Given that the majority of RCTs only evaluated the efficacy of PNS after a single session, we elected to extract data pertaining to outcomes immediately following the initial stimulation session when studies involved multiple sessions to promote consistency across the analyzed data.

If the means (*m*) and standard deviation (*SD*) we needed were not provided in the original article and the original data could not be obtained from the authors, we used the method of Hozo to estimate *m* and *SD* (Hozo et al., 2005). By using the sample size, median (*M*), maximum value (*a*), minimum value (*b*), and the difference value between maximum and minimum (*R*) provided in the studies, we calculated the corresponding *m* and *SD* using relevant formulas. When the sample size was less than or equal to 25, the formula for calculating the mean was $\frac{a+2M+b}{4}$; when the sample size was greater than 25, *m* = *M*, meaning the mean could be represented by the median. When the sample size was less than 15, the formula for calculating the *SD* was $\sqrt{\frac{{\left(b-a\right)}^{2}+{\frac{\left(a-2m+b\right)}{4}}^{2}}{12}}$; when the sample size was between 15 and 70, the formula for calculating the *SD* was $\frac{R}{4}$; when the sample size was greater than 70, the formula for calculating the *SD* was $\frac{R}{6}$. When median and interquartile range (IQR) values were not reported, the relevant data were extracted from figures by Web Plot Digitizer. Then the standard deviation of the paired differences between baseline (*T*_0_) and last follow‐up (*T*_1_) was estimated using the formula (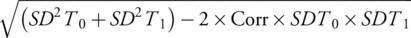) (Hozo et al., 2005).

### Risk of bias assessment

The risk of bias for each study was evaluated using the Revised Cochrane Risk of Bias Tool (RoB 2.0) (Higgins et al., 2023). RoB 2.0 was structured into a domain‐based evaluation approach, allowing for a more detailed assessment of potential biases in five domains. The domains included: (1) randomization process, (2) deviations from the intended interventions, (3) missing outcome data, (4) measurement of the outcome, and (5) selection of the reported result. We employed RoB 2.0 to assess the types of bias within the meta‐analysis to evaluate the overall level of bias, and ensure the credibility of the study results. The risk bias of the included studies was classified into three levels as “low risk”, “some concerns” and “high risk” based on the results of the bias analysis for each domain.

### Statistical analysis

We conducted a meta‐analysis using Review Manager (RevMan) V.5.4 (The Nordic Cochrane Centre, The Cochrane Center, The Cochrane Collaboration, 2020, Copenhagen, Denmark) to analyze the outcomes and effect measures (mean difference) in the synthesis or presentation. We used the inverse variance (I‐V), standardized mean difference (SMD), and 95% CI (confidence interval) to analyze the primary (perceived pain intensity and analgesic consumption) and secondary outcome data (ROM and LOH). Outcome effects were calculated to assess the confidence in the effect estimate of the outcome and evaluated using a two‐tailed test with a significance level of .05 to determine the inconsistency index (*I* ^*2*^). To assess the treatment effects of specific types of PNSs and identify sources of heterogeneity, subgroup analyses were performed based on different types of PNSs. The study was deemed heterogeneous when *I* ^*2*^ exceeded 50% and highly heterogeneous when it exceeded 75%. Conversely, the study was considered homogeneous when *I* ^*2*^ was below 25%. In addition, heterogeneity across studies was further estimated using 95% prediction intervals (PIs) to estimate the uncertainty and variability of the results across included studies. The 95% PI for each outcome parameter was computed directly from effect sizes and standard errors reported in individual studies where available (Schwarzer et al., 2015; Spineli & Pandis, 2020). Alternatively, relevant formulas were applied to indirectly calculate 95% PIs when not explicitly provided (Higgins et al., 2023; Hozo et al., 2005). Based on a relatively large absolute difference between the estimated studies effects and 95% CI shown in the forest plots, we expected that the intervention effects are unlikely to be identical across studies. To avoid bias in the effect size estimates due to heterogeneity and enhance the persuasiveness of the research outcomes, the random effects model was performed (Borenstein et al., 2010; Caldwell et al., 2005; DerSimonian & Laird, 1986; Fleiss & Gross, 1991).

To correlate the probability of publication with effect size in the study outcomes, we scrutinized the potential publication bias by generating funnel plots. Eggers' rank correlation test combined with funnel plots were used to assess the evidence of publication bias by Stata V.16 (StataCorp LP, College Station, TX, USA). Moreover, sensitivity analyses were performed to evaluate the robustness of the results and to explore potential sources of heterogeneity or bias within the included studies. Individual studies were systematically excluded from the meta‐analyses one at a time, followed by recalculation of the summary effect. An influential study was considered a study whose removal changed the magnitude of the pooled effect by >10%.

### Grading the evidence

To evaluate the quality of evidence, the included studies were assessed using the Grading of Recommendations Assessment, Development and Evaluation (GRADE) approach (Kirmayr et al., 2021). The GRADE framework was applied to rate the certainty of the evidence for each outcome, considering several aspects such as risk of bias, inconsistency, indirectness, imprecision, publication bias and other upgraded considerations. The evidence was then categorized into high, moderate, low, or very low, to indicate the confidence levels in the findings.

## RESULTS

### Study selections and study characteristics

On September 29, 2023, a total of 1138 articles were retrieved from three basic databases through a comprehensive search: 87 from PubMed, 572 from Embase, and 479 from Cochrane Library. After removing duplicate records (195 studies) and non‐RCTs (215 studies), a remaining pool of 728 studies was obtained. Then, a second screening was applied to exclude studies that did not pertain to orthopedic operations (456 studies), PNS (104 studies), lower‐limb (100 studies), or adult studies (40 studies), leaving 28 articles. The full texts of 24 studies were selected in the third filtering process, of which 16 were subsequently excluded due to criteria such as the absence of required outcomes (nine studies), non‐English language (three studies), or lack of original research data (four studies). Ultimately, a total of eight studies were deemed eligible for inclusion in the meta‐analysis (Figure 1).

The detailed characteristics of the included studies are shown in Table 1. A total of eight studies enrolling 633 participants (321 in the experimental groups and 312 in the control groups) from five countries (America, four studies; Asia, three studies [Turkey, one study; Iran, one study and Israel, one study]; Europe, one study [Belgium, one study]) were analyzed. These clinical investigations spanned a considerable period, from 1990 to 2021, with the peak number of publications occurring in 2019, accounting for three studies (37.5%). Except for three studies that did not report gender ratios, the majority of articles exhibited a higher proportion of female participants than male participants.

**TABLE 1:** Characteristics of the included studies

No.	Author (year)	Effect (Y/N)	Intervention	Indication	Country	Groups	Age	(M/F)	Main outcome parameters
1	Kadı et al. (2019)	N	TES	TKA	Turkey	G1: active TES (*n* = 49) G2: sham TES (*n* = 49)	N/A	9/99	Pain intensity (VAS); 2. paracetamol intake; 3. ROM
2	Beckwée et al. (2018)	N	TENS	TKA	Belgium	G1: active TENS (*n* = 25) G2: sham TENS (*n* = 28)	N/A	19/34	Pain intensity (VAS); 2. analgesic consumption: opioid and nonopioid; 3. ROM
3	Angulo and Colwell (1990)	N	TENS	TKA	USA	G1: active TENS+CPM (*n* = 18) G2: sham TENS+CPM (*n* = 12)	N/A	20/28	Pain intensity (VAS): the percentage decrease in pain; 2. medication intake; 3. ROM; 4. LOH
4	Elboim‐Gabyzon et al. (2019)	Y	TENS	Hip fracture surgery	Israel	G1: active TENS (*n* = 23) G2: sham TENS (*n* = 18)	≧50	9/32	Pain intensity (NRS): rest, overnight, walk; 2. analgesics consumption: nonnarcotic and narcotic
5	Forogh et al. (2019)	N	TENS	ACL	Iran	G1: active TENS (*n* = 35) G2: sham TENS (*n* = 35)	[18,45]	N/A	Pain intensity (VAS); 2. ROM
6	Ilfeld (2021)	Y	PPNS	Ankle surgery or ACL	USA	G1: active PPNS (*n* = 31) G2: sham PPNS (*n* = 34)	N/A	N/A	Pain intensity (NRS): average, worst, defense; 2. the pain inference with physical and emotional functions; 3. analgesic consumption
7	Rakel et al. (2014)	Y	TENS	TKA	USA	G1: active TENS (*n* = 122) G2: sham TENS (*n* = 124)	[40,90]	145/173	Pain intensity (VAS): rest, movement, extension, flexion, gait; 2. pain sensitivity: pressure, heat pain; 3. analgesics consumption: morphine (opioid) and acetaminophen (nonopioid); 4. ROM
8	Walker et al. (1991)	N	TENS	TKA	USA	G1: active TENS+CPM (*n* = 18) G2: sham TENS+CPM (*n* = 12)	G1: [61,69] G2: [60,86]	N/A	Analgesics consumption; 2. ROM; 3. LOH

*Note*: Y, yes; N, no (whether PNS in the included literature has a beneficial effect on post‐surgery outcomes).

Abbreviations: ACL, anterior cruciate ligament; CPM, continuous passive motion; F, female; LOH, length of hospitalization; M, male; N/A, not applicable; NRS, numeric rating scale; PPNS, peripheral percutaneous nerve stimulation; ROM, range of motion; TENS, transcutaneous electrical nerve stimulation; TES, transcutaneous electrical stimulation; TKA, total knee arthroplasty; VAS, visual analog scale.

The predominant surgical procedure among the included clinical studies was total knee arthroplasty (five studies), while the other procedures encompassed hip fracture surgery (one study), anterior cruciate ligament (ACL) (one study), and ankle surgery or ACL (one study). In terms of PNS, TENS was utilized in six studies for peripheral nerve modulation, with only two studies demonstrating therapeutic effects on pain relief: pain intensity (Elboim‐Gabyzon et al., 2019; Rakel et al., 2014) and analgesic reduction. One study using TES did not exhibit significant therapeutic effects, while another study using PPNS demonstrated significant clinical benefits to analgesic relief (Ilfeld et al., 2021). Pain intensity assessment was included as an outcome measure in each clinical study, with most articles choosing analgesic consumption and ROM as clinical endpoints. Only two clinical studies incorporated LOH as one of the clinical outcome measures.

### Risk of bias

**FIGURE 2:**
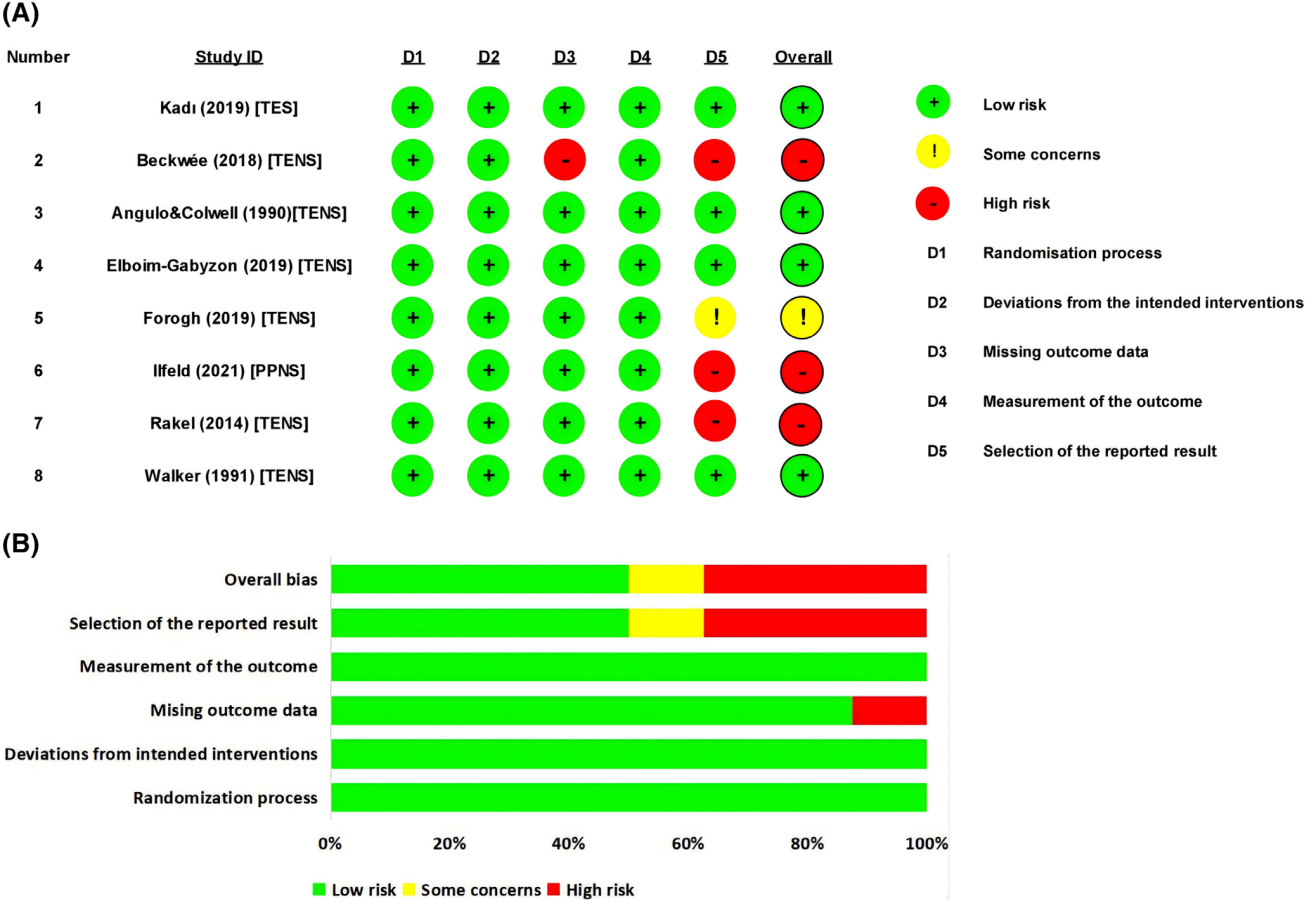
Risk of bias for each included trial assessed using RoB 2.0. A, risk of bias assessment for each included study. B, assessments made by review authors for each risk of bias domain are presented as percentages across all included studies. RoB 2.0 was used to examine the suitability of the method used based on five domains including bias derived from the randomization process (D1), bias due to deviations from intended interventions (D2), bias due to missing outcome data (D3), bias in the measurement of the outcome (D4), and bias in the selection of the reported result (D5). TENS, transcutaneous electrical nerve stimulation; PPNS, peripheral percutaneous nerve stimulation; TES, transcutaneous electrical stimulation. Green circles and green bars, the low risks of bias; yellow circles and yellow bars, some concerns of bias; red circles and red bars, high risks of bias.

Figure 2 shows the risk of bias results assessed across five domains using RoB 2.0 for each study included in the meta‐analysis (Higgins et al., 2023). In the included RCTs, all studies (100%) reported adequate randomization processes, had no deviations from the intended intervention, and blinded the outcome assessors. Seven studies (87.5%) were free of missing outcome data (only one reported incomplete outcome data because of a high proportion of loss to follow‐up) and four studies (50%) had reported all planned outcomes (three studies showed high risk and one showed some concerns for selection of reported results). Of the eight included trials, four studies had a low risk of bias, one study had a moderate risk of bias, and three had a high risk of bias. By summarizing the reasons for the high‐risk factors of domain 3 (missing outcome data) and domain 5 (selection of the reported result) in RoB 2.0, we found that a large amount of clinical data was missing, resulting in an inability to calculate clinical outcomes or a high proportion of dropouts. Additionally, the use of multiple acceptable outcome indicators for collecting clinical endpoint data simultaneously (such as different time points for pain intensity without a unified standard) and the application of various statistical methods for analyzing the same clinical endpoint were identified as fundamental causes of high bias risks in this study.

### Effect of PNS on pain relief

To identify whether PNS could decrease pain intensity scores, we included six studies with full clinical pain data in the analysis, comprising four using TENS, one using PPNS, and one using TES, with 285 subjects in the experimental groups and 288 subjects in the control groups.

**FIGURE 3:**
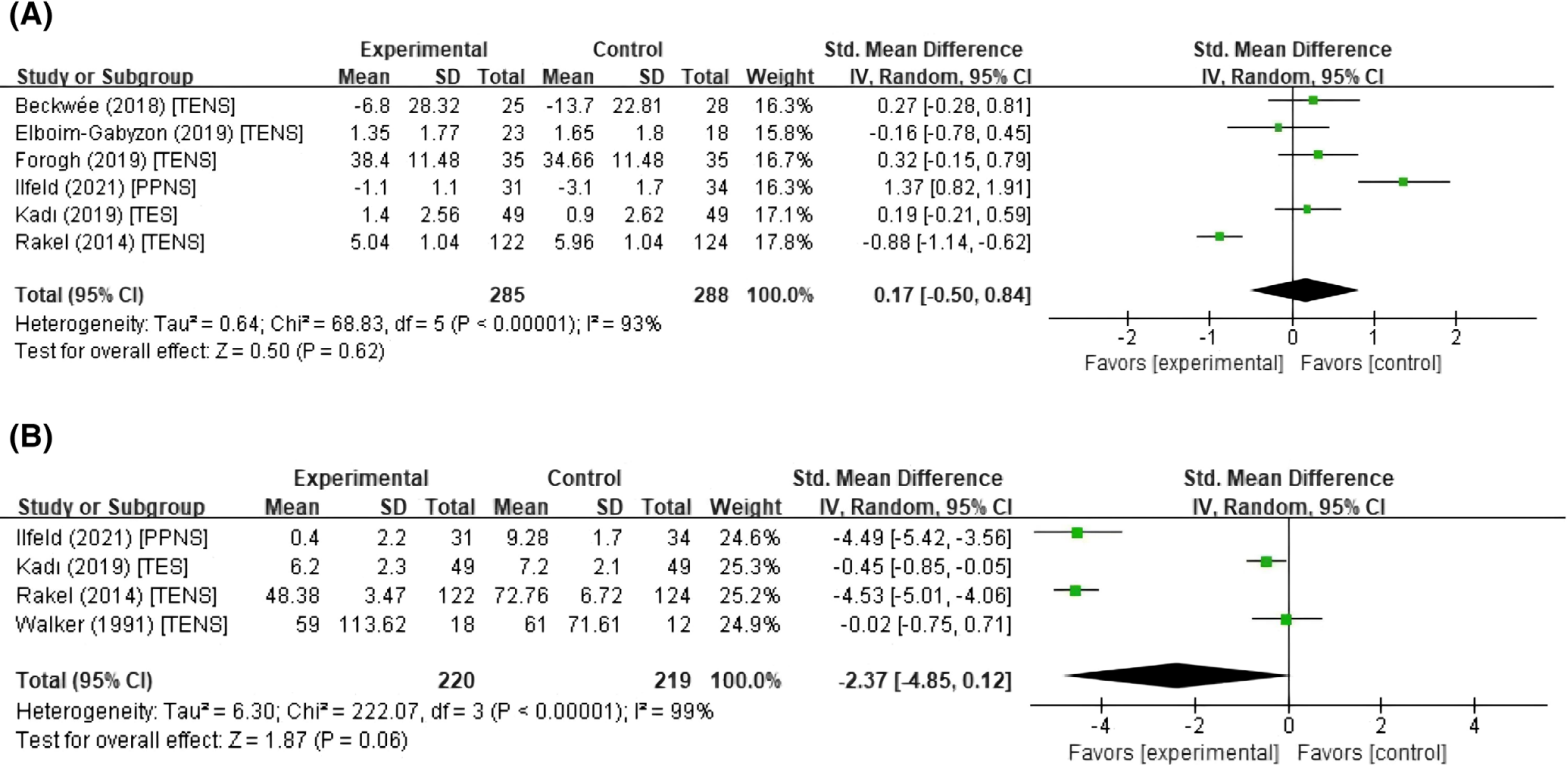
Forest plots of the studies assessing the pain relief effect of PNS. The results showed that PNS has no significant effect on pain intensity (A) but has a marginally significant effect on analgesic consumption (B) reduction during postoperative rehabilitation of the lower‐limb compared with the control groups. The intervention method of each study is presented following the respective study. SD, standard deviation; PNS, peripheral nerve stimulation; TENS, transcutaneous electrical nerve stimulation; PPNS, percutaneous peripheral nerve stimulation; TES, transcutaneous electrical stimulation; CI, confidence interval.

The forest plot depicting the results of the pooled and subgroup meta‐analysis is presented in Figure 3A and Supplementary File 5 (Figure S5A), respectively. The pooled pain relief effect of PNSs was not statistically significant, with high heterogeneity (*SMD*, 0.17; 95% CI, −0.50 to 0.84; *p* = .618; *I* ^2^ = 93%). The 95% PI for pain intensity ([−2.24, 2.58] included zero, Supplementary File 7) also demonstrated highly heterogeneous effects across studies. The results suggested that PNS did not decrease the intensity of pain in the recovery of orthopedic surgery compared to sham PNS.

### Effect of PNS on analgesic consumption

The meta‐analysis included four studies, comprising 220 subjects in the experimental groups and 219 subjects in the control groups, that provided complete numerical data on analgesic consumption before and after the intervention.

The forest plot depicting the results of the pooled and subgroup meta‐analysis is presented in Figure 3B and Supplementary File 5 (Figure S5B), respectively. The meta‐analysis revealed a marginal yet statistically significant reduction in analgesic consumption (*SMD*, −2.37; 95% CI, −4.85 to 0.12; *p* = .062; *I* ^2^ = 99%). The 95% PI ([−18.79, 13.07], Supplementary File 7) and *I* ^*2*^ (99%, *p*‐heterogeneity < .001) for analgesic consumption revealed highly heterogeneous effects across studies. These results indicated that PNS may have potential to improve pain relief and reduce the risk of complications associated with analgesic usage in the recovery of orthopedic surgery compared to sham PNS.

### Effect of PNS on functional improvements

**FIGURE 4:**
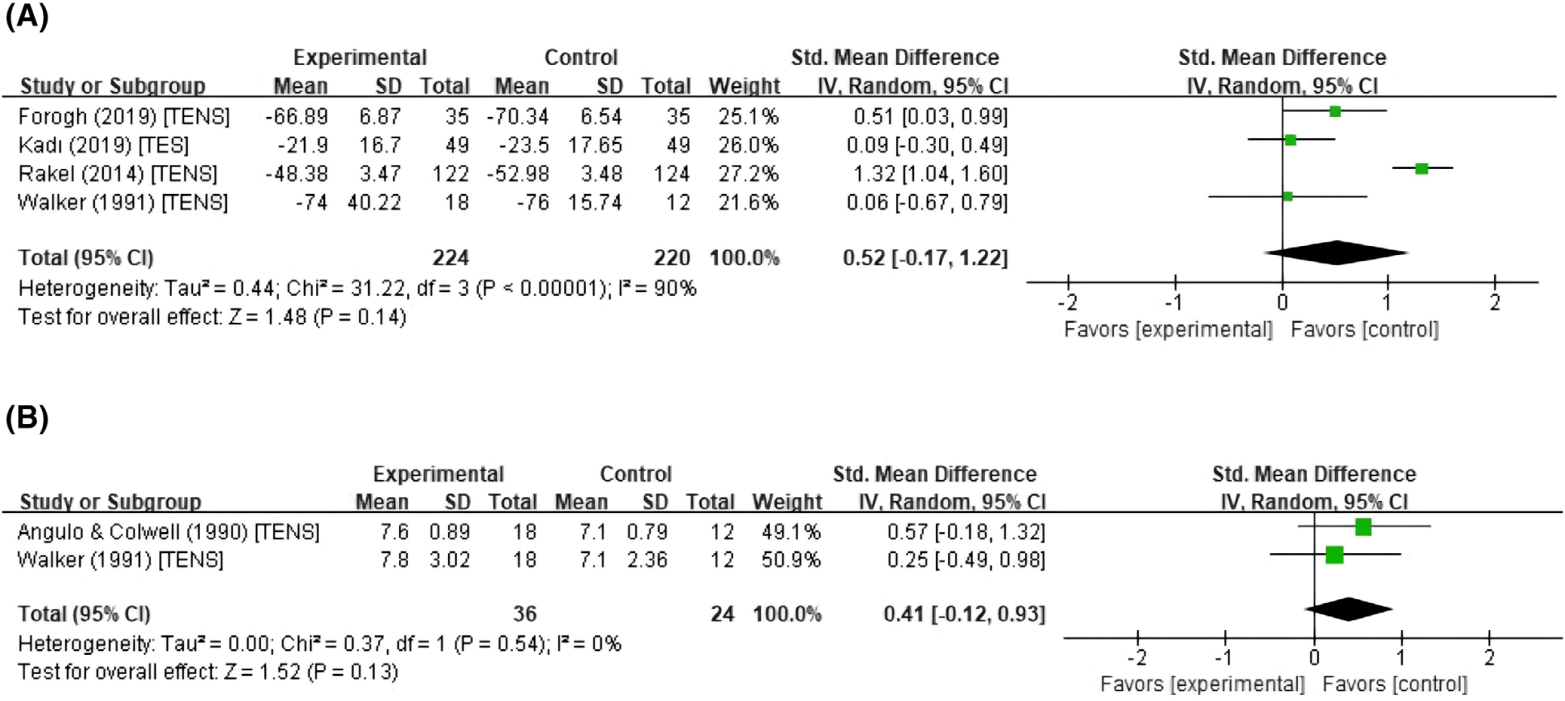
Forest plots of the studies assessing the functional improvement effect of PNS. The results showed that PNS has no significant effect on functional improvement for both the ROM (A) and LOH (B) in postoperative rehabilitation of the lower‐limb compared with the control groups. The intervention method of each study is presented following the respective study. ROM, range of motion; LOH, length of hospitalization; SD, standard deviation; PNS, peripheral nerve stimulation; TENS, transcutaneous electrical nerve stimulation; TES, transcutaneous electrical stimulation; CI, confidence interval.

Four studies, including 224 participants in the experimental groups and 220 in the control groups, provided specific data on ROM before and after the intervention. The forest plot depicting the results of the pooled and subgroup meta‐analysis is presented in Figure 4A and Supplementary File 6 (Figure S6). No improvement in ROM was found for PNS treatment compared to sham PNS (*SMD*, 0.52; 95% CI, −0.17 to 1.22; *p* = .140; *I* ^2^ = 90%). The 95% PI [−2.71, 3.76] and the value of *I* ^2^ (90%, *p‐*heterogeneity < .001) for ROM revealed highly heterogeneous effects across studies (Supplementary File 7). Two studies, comprising 36 subjects in the experimental groups and 24 in the control groups, analyzed LOH (Angulo & Colwell, 1990; Walker et al., 1991). The forest plot depicting the results of the meta‐analysis is presented in Figure 4B (pooled analysis). The meta‐analysis revealed that PNS did not have an improvement in LOH compared to sham PNS (*SMD*, 0.41; 95% CI, −0.12 to 0.93; *p* = .129; *I* ^*2*^ = 0%). Although the 95% PI for the LOH cannot be computed due to the inclusion of only two RCT studies (Supplementary File 7), the value of *I* ^2^ (0%, *p*‐heterogeneity = .540) showed no heterogeneity in results. These results indicated that PNS has no significantly additional positive effect on functional improvements for both ROM and LOH in the recovery of orthopedic surgery compared to sham PNS.

### Publication bias

Due to an insufficient number of trial comparisons (*n* < 10), the symmetry of funnel plots for four outcome measures (pain intensity, analgesic consumption, ROM, and LOH) could not reliably assess publication bias (Supplementary File 8). The Egger test values of the four funnel plots were greater than 0.05 (*p* = .200, .711, .287, and .530, respectively), which did not reveal any substantial evidence of publication bias. However, there were points at the lower end of the triangle of the funnel plot, and each research point in the plot was asymmetrical, which indicated that the research exhibited heterogeneity (Supplementary File 8).

### Sensitivity analyses

In the sensitivity analysis regarding pain intensity, only one study was found to significantly influence the pooled effect magnitude in the remaining studies. The exclusion of the study conducted by Rakel et al. (2014) resulted in statistically significant results for pain intensity (*SMD*, 0.40; 95% CI, −0.07 to 0.86; *p* = .092; *I* ^2^ = 76%; *p*‐heterogeneity = .002), leading to alterations exceeding 10% (Supplementary File 9). Additionally, in the sensitivity analysis of ROM, the removal of the Rakel et al. (2014) also substantially impacted the outcomes (*SMD*, 0.23; 95% CI, −0.05 to 0.51; *p* = .105; *I* ^2^ = 0%; *p*‐heterogeneity = .370) (Rakel et al., 2014). However, the other two outcome parameters, namely analgesic consumption and LOH, did not demonstrate any influence on heterogeneity by the included studies.

### GRADE assessment

The GRADE assessments were graded as very low for pain intensity due to imprecision (the wide confidence interval), risk of bias (no significant therapeutic effect difference between experimental and control groups), and inconsistency (*I* ^2^ = 93%, *p*‐heterogeneity < .001), as low for analgesic consumption due to imprecision (the wide confidence interval) and inconsistency (*I* ^2^ = 99%, *p*‐heterogeneity < .001), as moderate for ROM due to imprecision (the wide confidence interval), and as moderate for LOH due to imprecision (the wide confidence interval) (Supplementary File 10).

## DISCUSSION

In this meta‐analysis, we evaluated the effectiveness of PNS in postoperative rehabilitation compared to the sham PNS by assessing pain relief (reduction in pain intensity and analgesic consumption) and functional improvements (increase in ROM and decrease in LOH) in postoperative recovery. Our findings suggest that PNS might not significantly decrease pain intensity but have a marginally significant influence on analgesic reduction in patients undergoing lower‐limb orthopedic surgery, with high heterogeneity observed. Additionally, there was no evidence to show significant improvements in functional outcomes, such as increased ROM or decreased LOH, after PNS intervention.

### Effectiveness of PNS in postoperative rehabilitation

Our pain relief analysis revealed that several PNSs had no superior effects on postoperative pain after orthopedic surgical rehabilitation across different orthopedic procedures like joint arthroplasty, fracture surgery, etc. (Beaupre et al., 2013; Griffin et al., 2014), as measured by pain intensity and analgesic consumption. Consistently, Level I evidence for PNS has not been identified for inclusion in current orthopedic surgical analgesia guidelines (Chow et al., 2023; Sampognaro & Harrell, 2022). However, this should not be interpreted as evidence that PNS techniques are ineffective in treating postoperative pain, as previous studies have demonstrated their usefulness in reducing pain intensity and adverse effects associated with postoperative pain (Beaupre et al., 2013; Griffin et al., 2014). PNSs are widely used for clinical pain management (Chow et al., 2023), including headaches (Ellens & Levy, 2011; Magis et al., 2007; Urits et al., 2020), facial pain (Amin et al., 2008; Johnson et al., 2015), low back pain (Knotkova et al., 2021), and neuropathic pain (Gibson et al., 2017). Additionally, based on our analysis of analgesic consumption, it seems that PNSs could offer some benefits in reducing the need for analgesics following orthopedic surgery. The absence of a significant difference between the experimental and control groups in this analysis might be attributable to the limited number of studies available and each study's relatively small sample size. Hence, further extensive research is necessary to examine the efficacy of PNSs in analgesic reduction.

Although most studies (six out of eight) that investigated TENS as an intervention did not demonstrate pain relief efficacy, the current results are inconclusive regarding the precise effectiveness of TENS for pain relief. One possible reason for the limited effectiveness of TENS is the small sample size in RCTs, complicating outcome differentiation (Higgins et al., 2023). Additionally, TENS may only be effective for specific types of pain. Several clinical trials have demonstrated the analgesic effects of TENS on the spinal cord segments and the central nervous system (Gibson et al., 2019). At the spinal level, TENS has been found to activate receptor mediators (e.g., gamma‐aminobutyric acid [GABA] and glycine) thought to be involved in inhibiting nociceptive traffic, thus relieving primary pain (Maeda et al., 2007; Sabino et al., 2008). Additionally, TENS has been shown to alleviate movement‐associated pain (e.g., fibromyalgia) by inhibiting downstream nerve activity and modulating pain substances (Dailey et al., 2013, 2020; Noehren et al., 2015; Vance et al., 2021). Advancing research into pain processing mechanisms has elucidated that the human body generates pain responses to harmful stimuli, particularly when nociceptive C fibers induce central sensitization, leading to intense pain sensations (Denk et al., 2014; Menendez et al., 1996). TENS can disrupt the transmission of nociceptive signals by activating inhibitory neurons in the spinal dorsal horn, thereby producing analgesic effects (Melzack & Wall, 1965). Therefore, future studies on the effectiveness of TENS therapy should consider the specific etiology and treatments for pain and other factors, such as physical therapies, self‐management practices, and psychological conditions when evaluating its effectiveness in reducing pain.

It bears noting that PNS appears to exhibit a marginally significant effect on reducing analgesic consumption but not for pain intensity. A potential explanation is that while PNS did not substantially improve subjective pain experiences, it may have partially mitigated inflammation or other processes sufficient to moderately decrease analgesic requirements (Chen et al., 2018; Jahangirifard et al., 2018). However, given the marginal significance and low quality of evidence graded by GRADE for analgesic consumption, this effect should be interpreted cautiously and further research is very likely to have an important impact on our confidence in the precision of this estimate. Additionally, divergent mechanisms may involve subjective self‐reported pain intensity versus analgesic medication intake behaviors (Bannister et al., 2015; Mendell, 2014). Other factors such as patient expectations and prescriber practices may further influence analgesic utilization (Hu et al., 2018; Wood et al., 2022). Further research is warranted to elucidate the determinacy and mechanisms of discordant effects of PNS on pain intensity versus analgesic consumption.

For functional improvements, meta‐analysis results demonstrated no statistically significant difference in two functional outcomes, i.e., increased ROM and decreased LOH, between the experimental and control groups during postoperative rehabilitation. Similarly, previous studies on alternate measures of postoperative recovery produced inconclusive results regarding the effect of PNSs on postoperative recovery. While some RCTs have shown improvements in walking distance and greater mobility with active modulation (Arvidsson & Eriksson, 1986; Rakel et al., 2014; Walker et al., 1991), others have suggested that PNSs do not contribute to functional improvements (Angulo & Colwell, 1990; Forogh et al., 2019; Walker et al., 1991). The inconsistencies among studies may be attributed to the limited number of studies included and the small sample sizes used in RCTs.

### Investigation for reasons for high heterogeneity

Given the small sample size and high heterogeneity in the meta‐analysis, this study implied the reasons for the high heterogeneity effects of different types of PNSs in postoperative orthopedic rehabilitation of the lower limb. We considered the following sources of heterogeneity: (1) Variation in the types of PNSs and outcome parameters in RCTs, particularly regarding inconsistent pain intensity rating scales among the included studies—this inconsistency led to differences in reliability and validity, resulting in significant heterogeneity after analysis; (2) interactions between variables in RCTs were influenced by various complex factors, such as significant individual variations and stimulation parameters; (3) data loss and variations in the assessment of pain intensity at different time points hindered the ability of meta‐analysis to effectively summarize and analyze the data, leading to a high level of heterogeneity in the clinical study.

### Limitations and implications

This study has several limitations that should be considered. First, the treatment duration in the included studies was generally short, and the absence of preoperative evaluation data prevented a comparison with postoperative data, complicating the demonstration of specific improvements. Second, the reliability and validity of the analysis results might be compromised by various factors, such as the number of included articles, the small sample size of each clinical study, and incomplete treatment data in the articles. Third, the absence of a unified evaluation system restricted the ability to accurately determine the impact of the intervention on postoperative recovery. Finally, restricting this review to only English language publications presented an inherent language bias. Though the reviewed evidence incorporates geographic diversity, the omission of non‐English‐language studies might have resulted in precluding potentially relevant research published in other languages. Multilingual assessments in the future are required to fully encapsulate the global evidence base and reduce the risk of over‐generalization. Therefore, future studies should be designed more thoroughly, incorporating multiple stimulation parameters and conducting large‐sample, multi‐center, and well‐conducted RCTs with longer follow‐up periods to determine the efficacy of PNS and establish optimal clinical protocols for postoperative treatments of lower‐limb orthopedic surgery.

The study demonstrated PNS can relieve orthopedic postoperative pain to a certain extent (reducing analgesic consumption), deepening our understanding of its therapeutic effect. However, the limited sample sizes and high between‐study heterogeneity of the present studies suggest that additional high‐quality RCTs investigating PNS for orthopedic postoperative rehabilitation are warranted to provide more robust evidence to guide clinical implementation, as studies with longer follow‐up durations and clear reporting of stimulation parameters could help derive more solid conclusions.

## CONCLUSIONS

PNSs have no significant effect on pain relief and functional improvements but have a marginally significant effect on analgesic consumption reduction during postoperative rehabilitation of the lower limb. The reliability and validity of these results have been limited due to several potential confounding factors, such as a limited sample size, high between‐study heterogeneity, and wide confidence intervals observed in the included studies. Future studies should be conducted with increased sample sizes, extended follow‐up periods, and a wider range of stimulation parameters, to identify the key factors contributing to the success of adding PNS in the treatment of orthopedic postoperative patients.

## FUNDING INFORMATION

The study is supported by the National Natural Science Foundation of China (No. 32200901, 32171078), the project funded by China Postdoctoral Science Foundation (No. 2022M723363), and the Scientific Foundation of Institute of Psychology, Chinese Academy of Sciences (No. E2CX6815CX).

## CONFLICT OF INTEREST STATEMENT

The authors declare there are no competing interests.

## ETHICS STATEMENT

This work was secondary research (a systematic review) and did not involve the use of human subjects; thus, there were no ethical considerations.

## Supporting information


**Data S1.** Supporting Information.

## Data Availability

All data relevant to the study are included in the article or uploaded as supplementary information. Extracted data are available on request to the corresponding author.
