# Supplementary material for: Peripheral nerve stimulation for lower‐limb postoperative recovery: A systematic review and meta‐analysis of randomized controlled trials
Source: Psych J. 2024 Sep 16;14(1):15–27. doi: 10.1002/pchj.794 (PMC11787885; doi:10.1002/pchj.794)
Supplement: Supplementary file 1 — Data S1. Supporting Information. [file PCHJ-14-15-s001.docx]

**Online Supplemental Information**

APPENDIX

| Supplementary | Title | Page |
| --- | --- | --- |
| File number |  | Number |
| 1 | PRISMA Checklist | Page 2-7 |
| 2 | AMSTAR 2 Checklist | Page 8-10 |
| 3 | PICOS framework of the search strategy | Page 11 |
| 4 | Search strategy | Page 12-17 |
| 5 | Subgroup analysis for the pain relief of included RCTs | Page 18-19 |
| 6 | Subgroup analysis for the functional improvements of included RCTs | Page 20 |
| 7 | Prediction interval for included RCTs | Page 21-22 |
| 8 | Funnel plot for the effect of included RCTs | Page 23 |
| 9 | Sensitivity analysis of the systematic removal of each RCT | Page 24-25 |
| 10 | GRADE assessment of study quality | Page 26-27 |

**Supplementary File 1. PRISMA Checklist.**


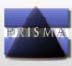


**PRISMA 2020 Checklist**

| **Section and Topic** | **Item #** | **Checklist item** | **Location where the item is reported** |
| --- | --- | --- | --- |
| **TITLE** | | |  |
| Title | 1 | Identify the report as a systematic review. | Page 1 |
| **ABSTRACT** | | |  |
| Abstract | 2 | See the PRISMA 2020 for Abstracts checklist. | Page 1 |
| **INTRODUCTION** | | |  |
| Rationale | 3 | Describe the rationale for the review in the context of existing knowledge. | Page 1-2 |
| Objectives | 4 | Provide an explicit statement of the objective(s) or question(s) the review addresses. | Page 2 |
| **METHODS** | | |  |
| Eligibility criteria | 5 | Specify the inclusion and exclusion criteria for the review and how studies were grouped for the syntheses. | Page 2-3, Figure 1, Supplementary File 3-4 |
| Information sources | 6 | Specify all databases, registers, websites, organizations, reference lists, and other sources searched or consulted to identify studies. Specify the date when each source was last searched or consulted. | Page 2-3, Supplementary File 3-4 |
| Search strategy | 7 | Present the full search strategies for all databases, registers, and websites, including any filters and limits used. | Page 2-3, Figure 1, Supplementary File 3-4 |
| Selection process | 8 | Specify the methods used to decide whether a study met the inclusion criteria of the review, including how many reviewers screened each record and each report retrieved, whether they worked independently, and if applicable, details of automation tools used in the process. | Page 3, Figure1, Supplementary File 3-4 |
| Data collection process | 9 | Specify the methods used to collect data from reports, including how many reviewers collected data from each report, whether they worked independently, any processes for obtaining or confirming data from study investigators, and if applicable, details of automation tools used in the process. | Page 3-4 |
| Data items | 10a | List and define all outcomes for which data were sought. Specify whether all results that were compatible with each outcome domain in each study were sought (e.g., for all measures, time points, analyses), and if not, the methods used to decide which results to collect. | Page 3-4 |
|  | 10b | List and define all other variables for which data were sought (e.g., participant and intervention characteristics, funding sources). Describe any assumptions made about any missing or unclear information. | Page 4 |
| Study risk of bias assessment | 11 | Specify the methods used to assess the risk of bias in the included studies, including details of the tool(s) used, how many reviewers assessed each study and whether they worked independently, and if applicable, details of automation tools used in the process. | Page 4 |
| Effect measures | 12 | Specify for each outcome the effect measure(s) (e.g., risk ratio, mean difference) used in the synthesis or presentation of results. | Page 4 |
| Synthesis methods | 13a | Describe the processes used to decide which studies were eligible for each synthesis (e.g., tabulating the study intervention characteristics and comparing against the planned groups for each synthesis (item #5)). | Page 3-4 |
|  | 13b | Describe any methods required to prepare the data for presentation or synthesis, such as handling of missing summary statistics, or data conversions. | Page 3-4 |
|  | 13c | Describe any methods used to tabulate or visually display the results of individual studies and syntheses. | Page 4 |
|  | 13d | Describe any methods used to synthesize results and provide a rationale for the choice(s). If meta-analysis was performed, describe the model(s), method(s) to identify the presence and extent of statistical heterogeneity, and software package(s) used. | Page 4 |
|  | 13e | Describe any methods used to explore possible causes of heterogeneity among study results (e.g., subgroup analysis, meta-regression). | Page 4 ; Supplementary File 5 |
|  | 13f | Describe any sensitivity analyses conducted to assess the robustness of the synthesized results. | Page 4 |
| Reporting bias assessment | 14 | Describe any methods used to assess the risk of bias due to missing results in a synthesis (arising from reporting biases). | Page 4 |
| Certainty assessment | 15 | Describe any methods used to assess certainty (or confidence) in the body of evidence for an outcome. | Page 4-5 |
| **RESULTS** | | |  |
| Study selection | 16a | Describe the results of the search and selection process, from the number of records identified in the search to the number of studies included in the review, ideally using a flow diagram. | Page 5, Figure 1 |
|  | 16b | Cite studies that might appear to meet the inclusion criteria, but which were excluded, and explain why they were excluded. | Page 5, Figure 1 |
| Study characteristics | 17 | Cite each included study and present its characteristics. | Page 5-6, Table 1 |
| Risk of bias in studies | 18 | Present assessments of risk of bias for each included study. | Page 6, Figure 2 |
| Results of individual studies | 19 | For all outcomes, present, for each study: (a) summary statistics for each group (where appropriate) and (b) an effect estimates and its precision (e.g., confidence/credible interval), ideally using structured tables or plots. | Page 7-8, Figure 3-4 |
| Results of syntheses | 20a | For each synthesis, briefly summarise the characteristics and risk of bias among contributing studies. | Page 7-8, Table 1, Figure 2-4 |
|  | 20b | Present results of all statistical syntheses conducted. If meta-analysis was done, present for each the summary estimate and its precision (e.g., confidence/credible interval) and measures of statistical heterogeneity. If comparing groups, describe the direction of the effect. | Page 7-8, Figure 3-4 |
|  | 20c | Present results of all investigations of possible causes of heterogeneity among study results. | Page 6-8, Figure 2-4, Supplementary File 5-10 |
|  | 20d | Present results of all sensitivity analyses conducted to assess the robustness of the synthesized results. | Page 8, Supplementary File 9 |
| Reporting biases | 21 | Present assessments of risk of bias due to missing results (arising from reporting biases) for each synthesis assessed. | Page 8, Supplementary File 8 |
| Certainty of evidence | 22 | Present assessments of certainty (or confidence) in the body of evidence for each outcome assessed. | Page 8, Supplementary File 10 |
| **DISCUSSION** | | |  |
| Discussion | 23a | Provide a general interpretation of the results in the context of other evidence. | Page 8-9 |
|  | 23b | Discuss any limitations of the evidence included in the review. | Page 10 |
|  | 23c | Discuss any limitations of the review processes used. | Page 10 |
|  | 23d | Discuss the implications of the results for practice, policy, and future research. | Page 10 |
| **OTHER INFORMATION** | | |  |
| Registration and protocol | 24a | Provide registration information for the review, including the register name and registration number, or state that the review was not registered. | Page 2 |
|  | 24b | Indicate where the review protocol can be accessed, or state that a protocol was not prepared. | Page 2 |
|  | 24c | Describe and explain any amendments to information provided at registration or in the protocol. | None |
| Support | 25 | Describe sources of financial or non-financial support for the review, and the role of the funders or sponsors in the review. | Page 10 |
| Competing interests | 26 | Declare any competing interests of review authors. | Page 10 |
| Availability of data, code, and other materials | 27 | Report which of the following are publicly available and where they can be found: template data collection forms; data extracted from included studies; data used for all analyses; analytic code; any other materials used in the review. | Page 10, Supplementary Files |

From: Page MJ, McKenzie JE, Bossuyt PM, Boutron I, Hoffmann TC, Mulrow CD, et al. *The PRISMA 2020 statement: an updated guideline for reporting systematic reviews.* BMJ 2021;372: n71. Doi: 10.1136/bmj.n71

For more information, visit: <http://www.prisma-statement.org/>


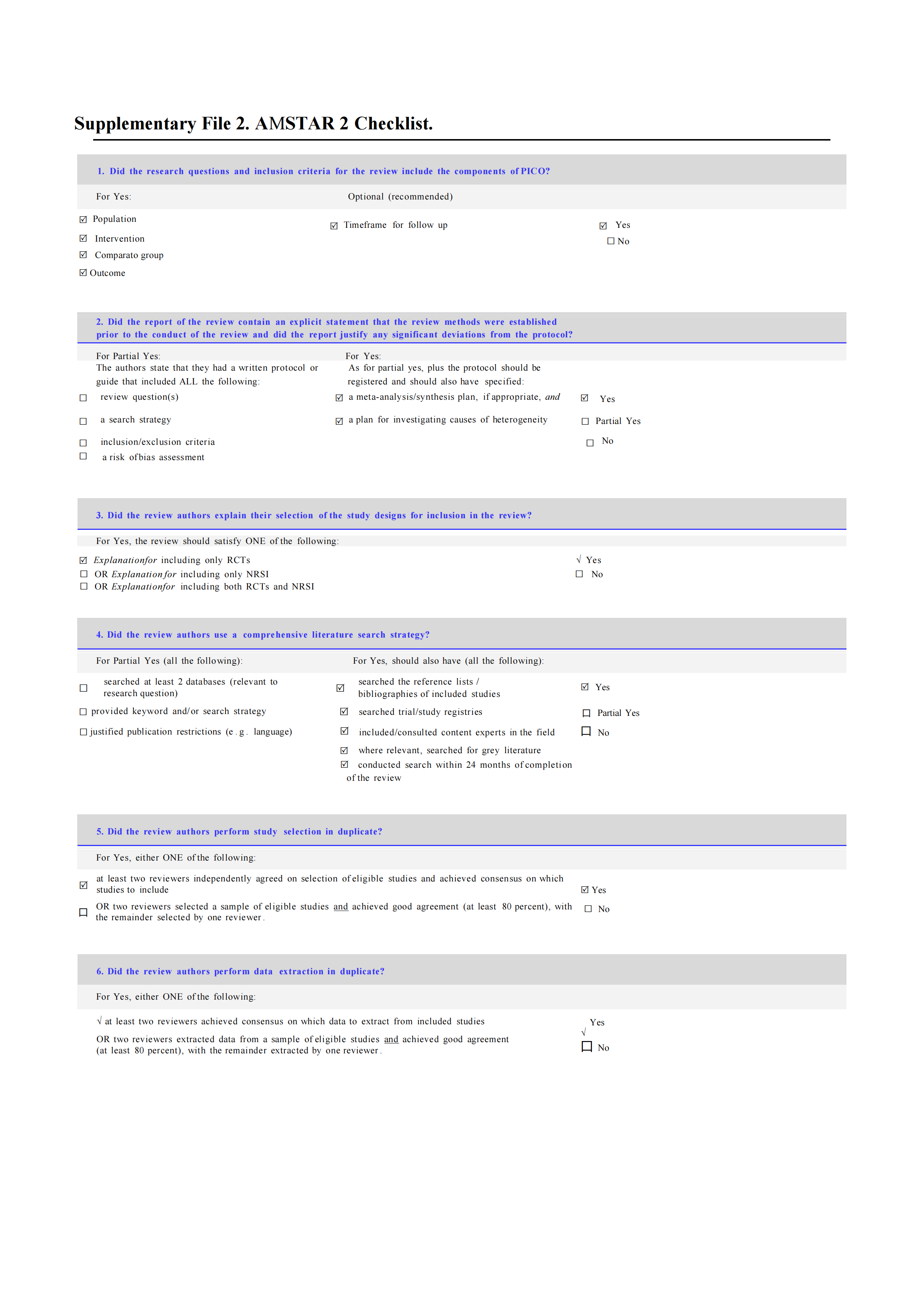


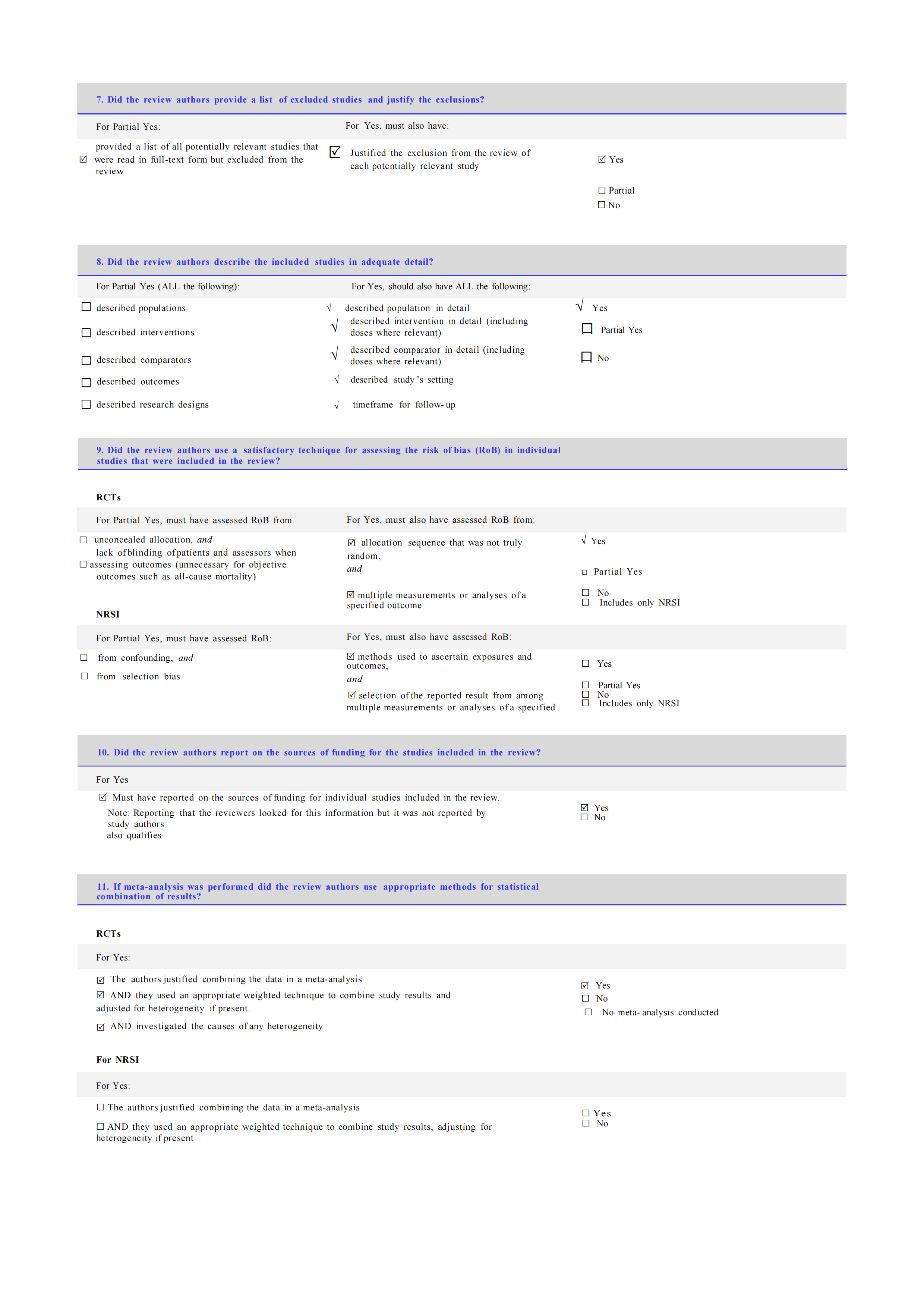


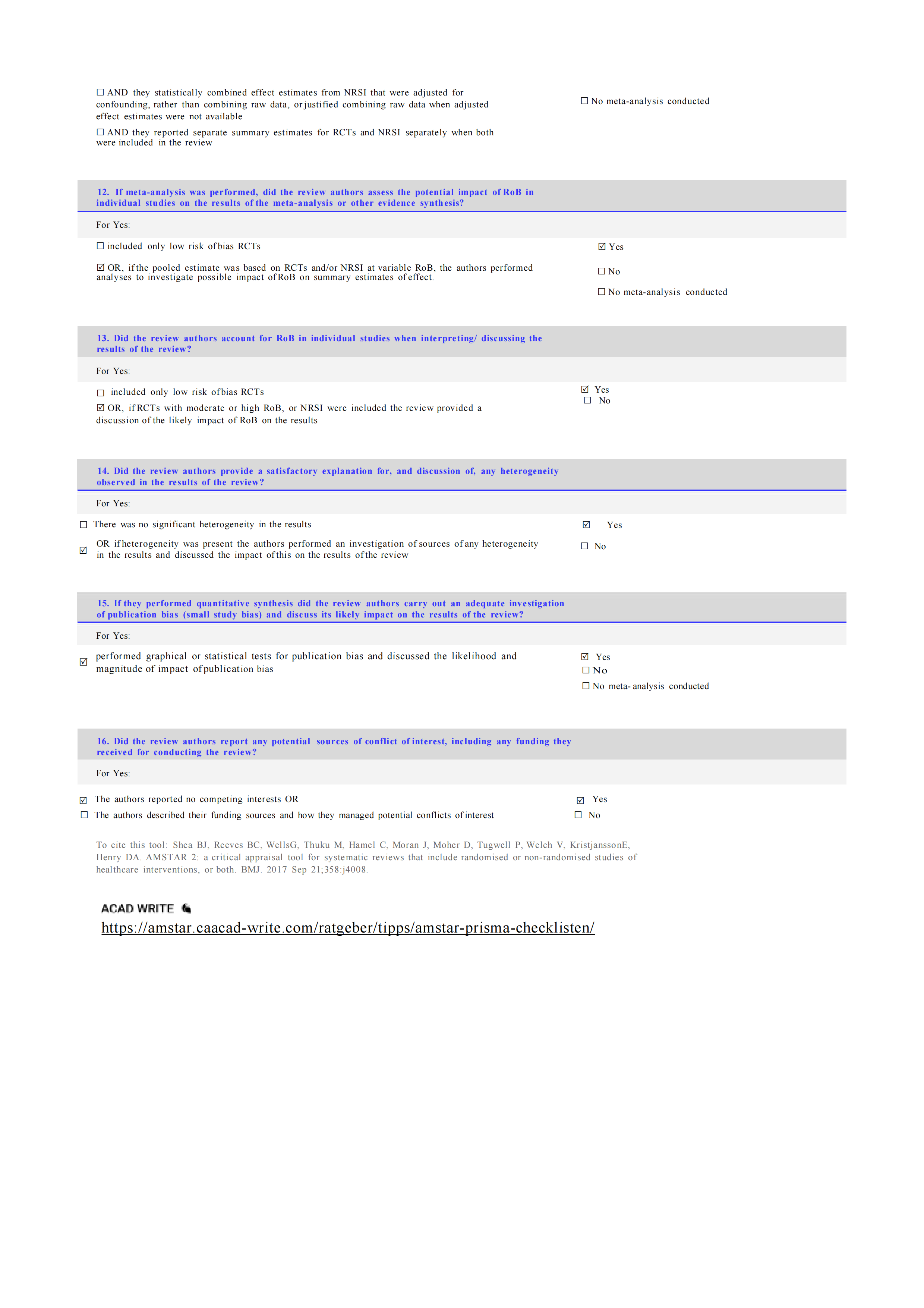


**Supplementary File 3. PICOS^a^ framework of the search strategy.**

| Participants (P) | Intervention (I) | Comparison (C) | Outcomes (O) | Study Design (S) |
| --- | --- | --- | --- | --- |
| Patients who underwent lower-limb orthopedic surgery | PNS, including TENS, PPNS, NES, ES, TEAS, and TES | The differences between the experimental group and the control group | The primary outcomes were pain intensity and analgesic consumption, while the secondary outcomes were ROM and LOH | RCTs (randomized controlled trials) |

^a^: Moher D, Shamseer L, Clarke M, Ghersi D, Liberati A, Petticrew M, Shekelle P, Stewart LA and PRISMA-P Group. *Preferred reporting items for systematic review and meta-analysis protocols (PRISMA-P) 2015 statement.* Systematic Reviews 2015; 4:1 https://systematicreviewsjournal.biomedcentral.com/articles/10.1186/2046-4053-4-1. PNS, peripheral nerve stimulation; TENS, transcutaneous electrical nerve stimulation; PPNS, peripheral percutaneous nerve stimulation; NES, nerve electric stimulation; ES, electric stimulation; TEAS, transcutaneous electrical acupoint stimulation; TES, transcutaneous electrical stimulation; ROM, range of motion; LOH, length of hospitalization.

**Supplementary File 4. Search strategy.**

A. PubMed database.

| No. | Search Details |
| --- | --- |
| #1 | "electric stimulation"[MeSH Terms] |
| #2 | (Electric* Stimulation*) OR (Stimulation*, Electric*)[All Fields] |
| #3 | #1 OR #2 |
| #4 | "electric stimulation therapy"[MeSH Terms] |
| #5 | ((((therap* electric* stimulation*) OR (Stimulation*, Therap* Electric)) OR (Electrotherap*)) OR (Interferential Current Electrotherapy)) OR (Electrotherap*, Interferential Current)[All Fields] |
| #6 | #4 OR #5 |
| #7 | "transcutaneous electric nerve stimulation"[MeSH Terms] |
| #8 | ((((((((((((((((Electric* Stimula*, Transcutaneous) OR (Percutaneous Electric* Nerve Stimulation)) OR (Transcutaneous Electric* Stimulation)) OR (Transdermal Electrostimulation)) OR (Transcutaneous Nerve Stimulation)) OR (TENS)) OR (Percutaneous Neuromodulation Therap*)) OR (Electric* Neuromodulation*, Percutaneous)) OR (Percutaneous Electrical Neuromodulations)) OR (Analgesic Cutaneous Electrostimulation)) OR (Electrostimulation, Analgesic Cutaneous)) OR (Electroanalgesia*)) OR (Trancutaneous electric* acupoint stimulation)) OR (PPNS)) OR (Percutaneous peripheral nerve stimulation)) OR (nerve electric* stimulation)) OR (TES)[All Fields] |
| #9 | #7 OR #8 |
| #10 | "vagus nerve stimulation"[MeSH Terms] |
| #11 | ((((Nerve Stimulat*, Vagus) OR (Vagus Nerve Stimulat*)) OR (Nerve Stimulation*, Vagal)) OR (Stimulation*, Vagal Nerve)) OR (Vagal Nerve Stimulation*)[All Fields] |
| #12 | #10 OR #11 |
| #13 | "implantable neurostimulators"[MeSH Terms] |
| #14 | (Implant* Neurostimulat*) OR (Implanted Nerve Stimulation Electrodes)[All Fields] |
| #15 | #3 OR #6 OR #9 OR #12 OR #13 OR #14 |
| #16 | "orthopedics"[MeSH Terms] OR "orthopedic procedures"[MeSH Terms] |
| #17 | ((((((((Orthopedic Procedure*) OR (Procedure*, Orthopedic)) OR (Orthopedic Surgical Procedure*)) OR (Procedure*, Orthopedic Surgical)) OR (Surg* Procedure, Orthopedic)) OR (Orthopedic Surg*)) OR (Surg*, Orthopedic)) OR (Orthopedic Rehabilitation Surger*)) OR (Rehabilitation Surger*, Orthopedic)[All Fields] |
| #18 | #16 OR #17 |
| #19 | (((((((post operat*) OR (postoperat*)) OR (postsurg*)) OR (post surg*)) OR (afteroperat*)) OR (after operat*)) OR (aftersurg*)) OR (after surg*)[All Fields] |
| #20 | #18 OR #19 |
| #21 | Rehabilitation[MeSH Terms] |
| #22 | ((((rehabilitation) OR (habilitation)) OR (recover*)) OR (rehab*)) OR (habili*)[All Fields] |
| #23 | Enhanced Recovery After Surgery[MeSH Terms] |
| #24 | (Postsurgical Recover*, Enhanced)[All Fields] |
| #25 | #21 OR #22 OR #23 OR #24 |
| #26 | "randomized"[Title/Abstract] OR "placebo"[Title/Abstract] OR "clinical trials as topic"[MeSH Terms:noexp] OR "randomly"[Title/Abstract] OR "trial"[Title] NOT "animals"[MeSH Terms] NOT ("humans"[MeSH Terms] AND "animals"[MeSH Terms]) |
| #27 | #15 OR #20 OR #25 OR #26 |

1. EMBASE database.

| No. | Query |
| --- | --- |
| #1 | 'electrostimulation'/exp OR 'electrostimulation' |
| #2 | 'electric* stimulation*':ab,kw,ti OR 'stimulation*, electric*':ab,kw,ti |
| #3 | #1 OR #2 |
| #4 | 'nerve stimulation'/exp OR 'nerve stimulation' |
| #5 | 'vagus nerve stimulation'/exp OR 'vagus nerve stimulation' |
| #6 | 'spinal cord stimulation'/exp OR 'spinal cord stimulation' |
| #7 | 'sacral nerve stimulation'/exp OR 'sacral nerve stimulation' |
| #8 | 'neuromuscular electrical stimulation'/exp OR 'neuromuscular electrical stimulation' |
| #9 | 'transcranial electrical stimulation'/exp OR 'transcranial electrical stimulation' |
| #10 | 'brain depth stimulation'/exp OR 'brain depth stimulation' |
| #11 | 'electroacupuncture'/exp OR 'electroacupuncture' |
| #12 | 'transcranial magnetic stimulation'/exp OR 'transcranial magnetic stimulation' |
| #13 | 'transcutaneous electrical nerve stimulator'/exp OR 'transcutaneous electrical nerve stimulator' |
| #14 | #4 OR #5 OR #6 OR #7 OR #8 OR #9 OR #10 OR #11 OR #12 OR #13 |
| #15 | 'electrotherapy'/exp OR 'electrotherapy' |
| #16 | 'electroconvulsive therapy':ab,kw,ti OR 'heart stimulation':ab,kw,ti OR 'high frequency electrotherapy':ab,kw,ti OR 'low frequency electrotherapy':ab,kw,ti OR 'nerve stimulation':ab,kw,ti |
| #17 | 'electric* stimula*, transcutaneous':ab,kw,ti OR 'percutaneous electric* nerve stimulation':ab,kw,ti OR 'transcutaneous electric* stimulation':ab,kw,ti OR 'transdermal electrostimulation':ab,kw,ti OR 'transcutaneous nerve stimulation':ab,kw,ti OR 'stimulation, transcutaneous nerve':ab,kw,ti OR 'tens':ab,kw,ti OR 'percutaneous neuromodulation therapy':ab,kw,ti OR 'percutaneous neuromodulation therap*':ab,kw,ti OR 'electric* neuromodulation*, percutaneous':ab,kw,ti OR 'percutaneous electrical neuromodulations':ab,kw,ti OR 'analgesic cutaneous electrostimulation':ab,kw,ti OR 'electroanalgesia*':ab,kw,ti |
| #18 | 'therap* electric* stimulat*':ab,kw,ti OR 'stimulat*, therap* electric':ab,kw,ti OR 'electrotherap*':ab,kw,ti OR 'interferential current electrotherapy':ab,kw,ti OR 'electrotherap*, interferential current':ab,kw,ti |
| #19 | 'PPNS':ab,kw,ti OR 'TEAS':ab,kw,ti OR 'trancutaneous electric* acupoint stimulation':ab,ti,kw |
| #20 | #15 OR #16 OR #17 OR #18 OR #19 |
| #21 | #3 OR #14 OR #20 |
| #22 | 'orthopedic surgery'/exp OR 'orthopedic surgery' |
| #23 | 'cementoplasty':ab,kw,ti OR 'closed reduction procedure':ab,kw,ti OR 'fracture treatment':ab,kw,ti OR 'joint surgery':ab,kw,ti OR 'ligament surgery':ab,kw,ti OR 'open reduction procedure':ab,kw,ti OR 'osteotomy':ab,kw,ti OR 'tendon surgery':ab,kw,ti OR 'replacement surgery':ab,kw,ti |
| #24 | #22 OR #23 |
| #25 | 'post operat*':ab,kw,ti OR 'postoperat*':ab,kw,ti OR 'postsurg*':ab,kw,ti OR 'post surg*':ab,kw,ti OR 'afteroperat*':ab,kw,ti OR 'after operat*':ab,kw,ti OR 'aftersurg*':ab,kw,ti OR 'after surg*':ab,kw,ti |
| #26 | 'rehabilitation'/exp OR 'rehabilitation' |
| #27 | 'enhanced recovery after surgery'/exp OR 'enhanced recovery after surgery' |
| #28 | 'rehabilitation':ab,kw,ti OR 'habilitation':ab,kw,ti OR 'postsurgical recover*,enhanced':ab,kw,ti OR 'recover*,enhanced postsurgical':ab,kw,ti OR 'rehab*':ab,kw,ti OR 'hab*':ab,kw,ti |
| #29 | #26 OR #27 OR #28 |
| #30 | 'crossover procedure':de OR 'double-blind procedure':de OR 'randomized controlled trial':de OR 'single-blind procedure':de OR random*:de,ab,ti OR factorial*:de,ab,ti OR crossover*:de,ab,ti OR ((cross NEXT/1 over*):de,ab,ti) OR placebo*:de,ab,ti OR ((doubl* NEAR/1 blind*):de,ab,ti) OR ((singl* NEAR/1 blind*):de,ab,ti) OR assign*:de,ab,ti OR allocat*:de,ab,ti OR volunteer*:de,ab,ti |
| #31 | #21 AND #24 AND #25 AND #29 AND #30 |

C. Cochrane Central Register of Controlled Trials database.

| No. | Search Details |
| --- | --- |
| #1 | MeSH descriptor: [Electric Stimulation] explode all trees |
| #2 | (Electric* Stimulation*):ti,ab,kw OR (Stimulation*, Electric*):ti,ab,kw |
| #3 | #1 OR #2 |
| #4 | MeSH descriptor: [Electric Stimulation Therapy] explode all trees |
| #5 | (Therap* Electric* Stimulat*):ti,ab,kw OR (Therap* Electric* Stimulat*):ti,ab,kw OR (Electrotherap*):ti,ab,kw OR (Interferential Current Electrotherap*):ti,ab,kw OR (Electrotherap*, Interferential Current):ti,ab,kw |
| #6 | #4 OR #5 |
| #7 | MeSH descriptor: [Transcutaneous Electric Nerve Stimulation] explode all trees |
| #8 | (Electric* Stimula*, Transcutaneous):ti,ab,kw OR (Percutaneous Electric* Nerve Stimulation):ti,ab,kw OR (Percutaneous Neuromodulation Therap*):ti,ab,kw OR (Transdermal Electrostimulation):ti,ab,kw OR (Transcutaneous Nerve Stimulation):ti,ab,kw |
| #9 | (Electric* Neuromodulation*, Percutaneous):ti,ab,kw OR (Percutaneous Electrical Neuromodulation):ti,ab,kw OR (Analgesic Cutaneous Electrostimulation):ti,ab,kw OR (Electroanalgesia*):ti,ab,kw |
| #10 | (PPNS):ti,ab,kw OR (TEAS):ti,ab,kw OR (Trancutaneous electric* acupoint stimulation):ti,ab,kw OR (Nerve Stimulation* Vagas):ti,ab,kw |
| #11 | #7 OR #8 OR #9 OR #10 |
| #12 | #3 OR #6 OR #11 |
| #13 | (post-operat*):ti,ab,kw OR (postoperat*):ti,ab,kw OR (postsurg*):ti,ab,kw OR (post-surg*):ti,ab,kw OR (afteroperat*):ti,ab,kw (Word variations have been searched) |
| #14 | (after-operat*):ti,ab,kw OR (aftersurg*):ti,ab,kw OR (after-surg*):ti,ab,kw |
| #15 | #13 OR #14 |
| #16 | MeSH descriptor: [Rehabilitation] explode all trees |
| #17 | MeSH descriptor: [Enhanced Recovery After Surgery] explode all trees |
| #18 | (Rehab*):ti,ab,kw OR (hab*):ti,ab,kw OR (recover*):ti,ab,kw OR (recuperat*):ti,ab,kw |
| #19 | #16 OR #17 OR #18 |
| #20 | ("randomized-controlled trial"):ti,ab,kw OR ("placebo control trial"):ti,ab,kw OR ("randomized controlled studies"):ti,ab,kw OR ("randomized controlled clinical trial"):ti,ab,kw NOT ("animal*"):ti,ab,kw |
| #21 | #12 AND #15 AND #19 AND #20 |

ti, title; ab, abstract; kw, keyword.

**Supplementary File 5. Subgroup analysis for the pain relief of included RCTs.**

In the subgroup analysis of pain intensity, we categorized the different types of peripheral nerve stimulations (PNSs) into three subgroups, including transcutaneous electrical nerve stimulation (TENS) with 4 studies, transcutaneous electrical stimulation (TES) with 1 study and percutaneous peripheral nerve stimulation (PPNS) with 1 study. The results showed that the pain intensity relief effect of TENS was not significant, with high heterogeneity (SMD, -0.13; 95% CI, -0.82 to 0.56; *p* = .700; *I^2^* = 89%). PPNS showed a significant pain intensity relief effect, while TES showed no significant effect on pain relief. Heterogeneity was not adequately assessed due to the inclusion of only one clinical study in PPNS and TES (PPNS: SMD, 1.37; 95% CI, 0.82 to 1.91; *p* < .001, TES: SMD, 0.19; 95% CI, -0.21 to 0.59; *p* = .340, Figure S5A).

In the subgroup analysis of analgesic consumption, we classified different types of PNSs into three subgroups, namely TENS with 2 studies, PPNS with 1 study, and TES with 1 study. The results demonstrated that the effect of TENS on analgesic consumption was not statistically significant and showed high heterogeneity (SMD, -2.29; 95% CI, -6.71 to 2.14; *p* = .310; *I^2^* = 99%). However, both PPNS and TES showed significant reductions in analgesic consumption with no heterogeneity due to the inclusion of only one study (PPNS: SMD, -4.49; 95% CI, -5.42 to -3.56; *p* < .001; TES: SMD, -0.45; 95% CI, -0.85 to -0.05; *p* = .030, Figure S5B).

**A**


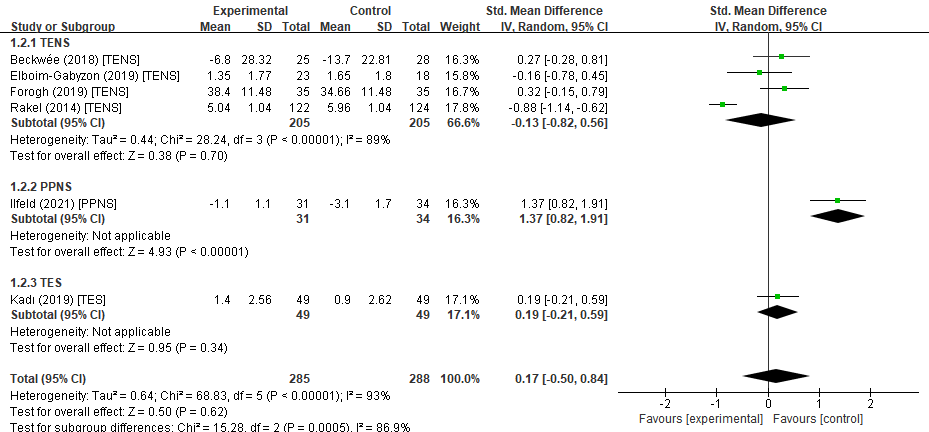


**B**


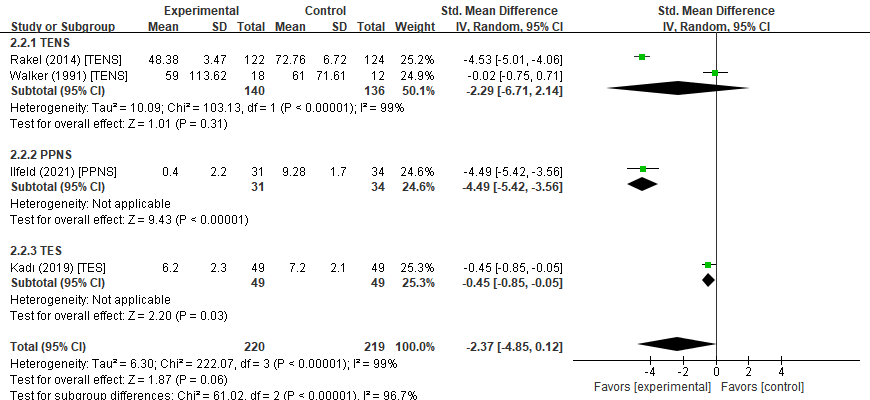


**Figure S5**. Forest plots for the studies assessing the pain relief effect of PNS in subgroup analysis. SD, standard deviation; PNS, peripheral nerve stimulation; TENS, transcutaneous electrical nerve stimulation; PPNS, percutaneous peripheral nerve stimulation; TES, transcutaneous electrical stimulation; CI, confidence interval.

**Supplementary File 6: Subgroup analysis for the functional improvements of included RCTs.**

In the subgroup analysis of ROM, we divided the various types of PNSs into two subgroups, namely TENS with 3 studies and TES with 1 study. The results indicated that the effect of TENS on ROM was not statistically significant, displaying high heterogeneity (SMD, 0.68; 95% CI, -0.07 to 1.44; *p* = .080; *I^2^* = 87%). Conversely, TES revealed no significant effect on ROM and lacked heterogeneity, which is attributable to the inclusion of only one study (SMD, 0.09; 95% CI, -0.30 to 0.49; *p* = .652, Figure S6).

Since both clinical studies incorporating the length of hospitalization (LOH) outcome measure utilized TENS as an intervention method and did not demonstrate significant therapeutic effects, no subgroup analysis was conducted.


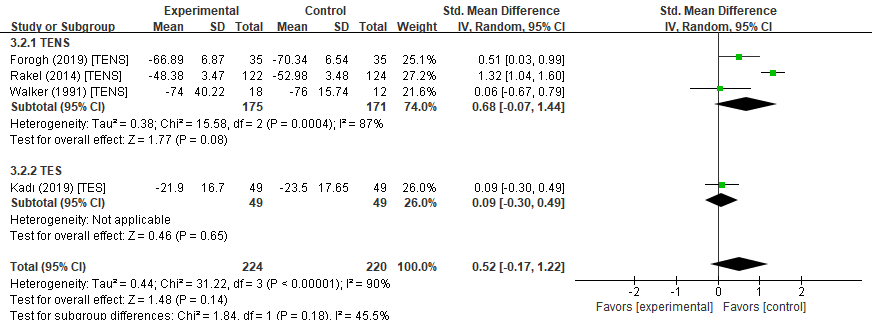


**Figure S6**. Forest plots for the studies assessing ROM of PNS in subgroup analysis. ROM, length of hospitalization; SD, standard deviation; PNS, peripheral nerve stimulation; TENS, transcutaneous electrical nerve stimulation; TES, transcutaneous electrical stimulation; CI, confidence interval.

**Supplementary File 7. Prediction interval of included trials.**

| **No.** | Study | Experimental Group | | | Control Group | | | ES | SE | 95% Prediction Interval |
| --- | --- | --- | --- | --- | --- | --- | --- | --- | --- | --- |
|  |  | Mean | SD | Sample | Mean | SD | Sample |  |  |  |
| **Pain Intensity** | |  |  |  |  |  |  |  |  |  |
| 1 | Beckwée (2018) [TENS] | -6.80 | 28.32 | 25 | -13.70 | 22.81 | 28 | 0.27 | 0.27 | [-2.24, 2.58] |
| 2 | Elboim-Gabyzon (2019) [TENS] | 1.35 | 1.77 | 23 | 1.65 | 1.80 | 18 | -0.17 | 0.31 |  |
| 3 | Forogh (2019) [TENS] | 38.40 | 11.48 | 35 | 34.66 | 11.48 | 35 | 0.32 | 0.24 |  |
| 4 | Ilfeld (2021) [PPNS] | -1.10 | 1.10 | 31 | -3.10 | 1.70 | 34 | 1.37 | 0.27 |  |
| 5 | Kadı (2019) [TES] | 1.40 | 2.56 | 49 | 0.90 | 2.62 | 49 | 0.19 | 0.20 |  |
| 6 | Rakel (2014) [TENS] | 5.04 | 1.04 | 122 | 5.96 | 1.04 | 124 | -0.88 | 0.13 |  |
| **Analgesic Consumption** | |  |  |  |  |  |  |  |  |  |
| 1 | IIfeld (2021) [PPNS] | 0.40 | 2.20 | 31 | 9.28 | 1.70 | 34 | -3.99 | 0.43 | [-18.79, 13.07] |
| 2 | Kadı (2019) [TES] | 6.20 | 2.30 | 49 | 7.20 | 2.10 | 49 | -0.43 | 0.20 |  |
| 3 | Rakel (2014) [TENS] | 48.38 | 3.47 | 122 | 72.76 | 6.72 | 124 | -7.00 | 0.34 |  |
| 4 | Walker (1991) [TENS] | 59.00 | 113.62 | 18 | 61.00 | 71.61 | 12 | -0.01 | 0.36 |  |
| **Range of Motion** | |  |  |  |  |  |  |  |  |  |
| 1 | Forogh (2019) [TENS] | -66.89 | 6.87 | 35 | -70.34 | 6.54 | 35 | 0.51 | 0.24 | [-2.71, 3.76] |
| 2 | Kadı (2019) [TES] | -21.90 | 16.70 | 49 | -23.50 | 17.65 | 49 | 0.09 | 0.20 |  |
| 3 | Rakel (2014) [TENS] | -48.38 | 3.47 | 122 | -52.98 | 3.48 | 124 | 1.32 | 0.14 |  |
| 4 | Walker (1991) [TENS] | -74.00 | 40.22 | 18 | -76.00 | 15.74 | 12 | 0.06 | 0.36 |  |
| **Length of Hospitalization** | |  |  |  |  |  |  |  |  |  |
| 1 | Angulo & Colwell (1990) [TENS] | 7.60 | 0.89 | 18 | 7.10 | 0.79 | 12 | 0.57 | 0.37 | n/a |
| 2 | Walker (1991) [TENS] | 7.80 | 3.02 | 18 | 7.10 | 2.36 | 12 | 0.25 | 0.36 |  |

E, experimental group; SD, standard deviation; ES, effect size; SE, standard error. TENS, transcutaneous electrical nerve stimulation; PPNS, peripheral percutaneous nerve stimulation; TES, transcutaneous electrical stimulation; n/a, not applicable or not available.

**Supplementary File 8. Funnel plot for the effect of included trials.**


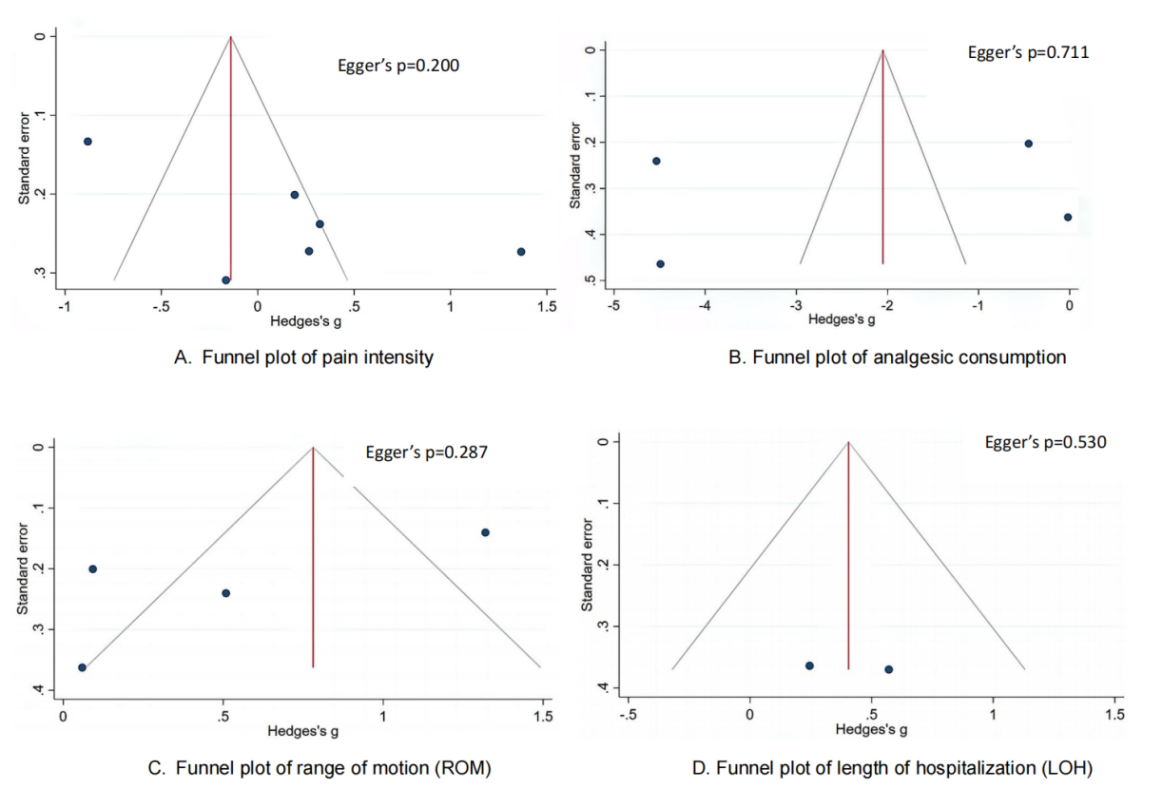


**Figure 8.** Funnel plot for the effect of included trials. The red solid line represents the overall pooled estimate for all studies included in the meta-analysis expressed as a weighted mean difference. The grey solid lines represent pseudo 95% confidence intervals (CIs). *p* values are derived from the quantitative assessment of publication bias by the Eggers' tests and Hedges' tests. The Eggers’ value of the four funnel plots is greater than 0.05, which did not reveal any substantial evidence of publication bias (Egger's *p* = .200, .711, .287, and .530, respectively). However, there were ports at the lower end of the triangle of the funnel plot and each research point in the plot was asymmetrical, indicating that the research exhibited heterogeneity.

**Supplementary File 9. Sensitivity analysis of the systematic removal of each trial*.**

| **Contents** | SMD [95% CI], *p*_1_-value  *I^2^*, *p*_2_-value | | | |
| --- | --- | --- | --- | --- |
|  | **Pain Intensity N=573** | **Analgesic Consumption N=409** | **Range of motion**  **N=374** | **Length of hospitalization**  **N=60** |
| All studies | 0.17 [-0.50, 0.84], *p*_1_ = .618 *I^2^* = 93%, *p*_2_ < .001 | -2.37 [-4.85, 0.12], *p*_1_ = .062  *I^2^* = 99%, *p*_2_ < .001 | 0.52 [-0.17, 1.22], *p*_1_ = .140 *I^2^* = 90%, *p*_2_ < .001 | 0.41 [-0.12, 0.93], *p*_1 =_ .129  *I^2^* = 0%, *p*_2_ = .542 |
| **Removal of** | | | | |
| Kadı (2019) [TES] | 0.17 [-0.66, 1.00], *p*_1_ = .689 *I^2^* = 94%, *p*_2_ < .001 | -3.01 [-5.97, -0.05], *p*_1_ = .046 *I^2^* = 98%, *p*_2_ < .001 | 0.68 [-0.07, 1.44], *p*_1_ = .077 *I^2^* = 87%, *p*_2_ < .001 | n/a |
| Beckwée (2018) [TENS] | 0.15 [-0.63, 0.93], *p*_1_ = .700 *I^2^* = 94%, *p*_2_ < .001 | n/a | n/a | n/a |
| Angulo & Colwell (1990) [TENS] | n/a | n/a | n/a | 0.25 [-0.49, 0.98], *p*_1_ = .510 n/a |
| Elboim-Gabyzon (2019) [TENS] | 0.24 [-0.54, 1.01], *p*_1_ = .553 *I^2^* = 94%, *p*_2_ < .001 | n/a | n/a | n/a |
| Forogh (2019) [TENS] | 0.14 [-0.65, 0.93], *p*_1_= .725  *I^2^* = 94%, *p*_2_ < .001 | n/a | 0.52 [-0.43, 1.47], *p*_1_ = .285 *I^2^* = 93%, *p*_2_ < .001 | n/a |
| Ilfeld (2021) [PPNS] | -0.07 [-0.64, 0.51], *p*_1_= .814 *I^2^* = 89%, *p*_2_ < .001 | -1.67 [-4.59, 1.24], *p*_1_ = .261  *I^2^* = 99%, *p*_2_ < .001 | n/a | n/a |
| Rakel (2014) [TENS] | 0.40 [-0.07, 0.86], *p*_1_ = .092 *I^2^* = 76%, *p*_2_ = .002 | -1.62 [-3.85, 0.61], *p*_1_ = .154  *I^2^* = 97%, *p*_2_ < .001 | 0.23 [-0.05, 0.51], *p*_1_ = .105 *I^2^* = 0%, *p*_2_ = .370 | n/a |
| Walker (1991) [TENS] | n/a | -3.15 [-6.20, -0.09], *p*_1_= .043  *I^2^* = 99%, *p*_2_ < .001 | 0.65 [-0.15, 1.45], *p*_1_= .111 *I^2^* = 93%, *p*_2_ < .001 | 0.57 [-0.18,1.32], *p*_1_ = .130 n/a |

*Sensitivity analysis included the removal of each single study from the meta-analyses one at a time and the summary effect was recalculated. An influential outlier was considered a study whose removal changed the magnitude of the pooled effect by >10%. *p*_1_-value, the heterogeneous effect of included studies; *p*_2_-value, the value of tests for overall effects; n/a, no substantial analysis; CI, confidence interval; SMD, standard mean difference; TENS, transcutaneous electrical nerve stimulation; PPNS, peripheral percutaneous nerve stimulation; TES, transcutaneous electrical stimulation.

**Supplementary File 10. GRADE assessment of study quality.**

| **Quality assessment** | | | | | | | **No. of patients** | | **Effect** | **Quality** | **Importance** |
| --- | --- | --- | --- | --- | --- | --- | --- | --- | --- | --- | --- |
|  |  |  |  |  |  |  |  |  |  |  |  |
| **No. of studies** | ***Design** | **Risk of bias** | **Inconsistency** | **Indirectness** | **Imprecision** | **Other considerations** | **Experimental Group** | **Control Group** | **Absolute** |  |  |
|  |  |  |  |  |  |  |  |  |  |  |  |
| **The Pain Intensity (follow-up mean 4 weeks; measured with: VAS (visual analogue scale); range of scores: 0-10; Better indicated by higher values)** | | | | | | | | | | | |
| 6 | Randomized trials | Serious ^a, d^ | Serious ^b^ | No serious indirectness | Serious ^c^ | None | 285 | 288 | SMD 0.17 higher (-0.50 lower to 0.84 higher) | ÅOOO | CRITICAL |
|  |  |  |  |  |  |  |  |  |  | VERY LOW |  |
| **The Analgesic Consumption (follow-up mean 4 weeks; measured with: the consumption of analgesia; Better indicated by higher values)** | | | | | | | | | | | |
| 4 | Randomized trials | No serious risk of bias | Serious ^b^ | No serious indirectness | Serious ^c^ | None | 220 | 219 | SMD -2.37 lower (-4.85 lower to 0.12 higher) | ÅÅOO | CRITICAL |
|  |  |  |  |  |  |  |  |  |  | LOW |  |
| **The Range of Motion (ROM) (follow-up mean 4 weeks; measured with: the goniometer; range of scores: 0-135; Better indicated by higher values)** | | | | | | | | | | | |
| 4 | Randomized trials | No serious risk of bias | No serious inconsistency | No serious indirectness | Serious ^c^ | None | 224 | 220 | SMD 0.52 higher (-0.17 lower to 1.22 higher) | ÅÅÅO | IMPORTANT |
|  |  |  |  |  |  |  |  |  |  | MODERATE |  |
| **The Length of Hospitalization (LOH) (follow-up mean 4 weeks; measured with: the day of participants in-hospital; Better indicated by higher values)** | | | | | | | | | | | |
| 2 | Randomized trials | No serious risk of bias | No serious inconsistency | No serious indirectness | Serious ^c^ | None | 36 | 24 | SMD 0.41 higher (-0.12 lower to 0.93 higher) | ÅÅÅO | IMPORTANT |
|  |  |  |  |  |  |  |  |  |  | MODERATE |  |

CI, Confidence interval; SMD, Standard mean difference; VAS, Visual analog scale; ROM, Range of motion; LOH, length of hospitalization. *Design – All outcomes started with high-quality evidence since all studies were randomized controlled trials. Risk of Bias – We rated down for risk of bias if most studies were considered to be at high risk of bias. Inconsistency– We assessed inconsistency using *I^2^* estimates where an *I^2^* =50%, *p* < .100 or higher indicates substantial heterogeneity. *I^2^* is the percentage of variability in the treatment estimates that was attributable to heterogeneity between studies. We rated down for inconsistency if there was substantial heterogeneity that was unexplained by any a priori sensitivity or subgroup analyses. Indirectness – We rated down for indirectness if there were factors relating to the participants, interventions, or outcomes that limited the generalizability of the results. Imprecision – We rated down for imprecision if the 95% confidence interval (95% CI) crossed the minimally important difference (MID) for harm. If the experimental group was compared with the control group, the treatment effect was not as good as the control group, resulting in negative effects.

1. Downgrade for risk of bias, as an experimental group was compared with the control group, the treatment resulted in no therapeutic effects.
2. Downgrade for inconsistency, as there was evidence of high heterogeneity which could not be explained.
3. Downgrade for impression, as the results had a wide confidence interval.

d. Downgrade for publication bias, as in clinical randomized controlled trials research, a large number of data related to respondents were lost.
